# Integrating molecular biomarkers in breast cancer rehabilitation. What is the current evidence? A systematic review of randomized controlled trials

**DOI:** 10.3389/fmolb.2022.930361

**Published:** 2022-09-08

**Authors:** Marco Invernizzi, Lorenzo Lippi, Arianna Folli, Alessio Turco, Lorenzo Zattoni, Antonio Maconi, Alessandro de Sire, Nicola Fusco

**Affiliations:** ^1^ Physical and Rehabilitative Medicine, Department of Health Sciences, University of Eastern Piedmont “A. Avogadro”, Novara, Italy; ^2^ Dipartimento Attività Integrate Ricerca e Innovazione (DAIRI), Translational Medicine, Azienda Ospedaliera SS. Antonio e Biagio e Cesare Arrigo, Alessandria, Italy; ^3^ Division of Pathology, IEO, European Institute of Oncology IRCCS, Milan, Italy; ^4^ Department of Oncology and Hemato-Oncology, University of Milan, Milan, Italy; ^5^ Physical and Rehabilitative Medicine Unit, Department of Medical and Surgical Sciences, University of Catanzaro “Magna Graecia”, Catanzaro, Italy

**Keywords:** breast cancer, biomarkers, rehabilitation, precision medicine, survivorship, quality of life

## Abstract

**Background:** Quality of life issues is a crucial burden in breast cancer (BC) survivors with relevant implications in terms of survivorship and health-care costs. The increasing long-term survival of these patients provides new challenges, with translational research now focusing on innovative and tailored approaches to improve their complex management. In this scenario, several emerging biomarkers have the potential to improve the clinical rehabilitative management of patients with BC. However, to date, guidelines supporting biomarker implementation in this area are still lacking. Therefore, the aim of this systematic review was to summarize the currently available biomarkers that might be potentially integrated into rehabilitation practice to promote a precision medicine approach to BC survivorship issues.

**Methods:** On 9th March 2022, PubMed, Scopus, Web of Science, Cochrane, and PEDro were systematically searched for randomized controlled trials (RCTs) assessing rehabilitation interventions in BC patients. Molecular biomarker modifications induced by physical exercise have been assessed through the review of the study protocols and published results. The Jadad scale was used to assess the quality of the studies included.

**Results:** Out of 2,224 records, 22 studies were included in the present systematic review. Exercise therapy showed significant results in 15 RCTs, in terms of metabolic biomarkers, including glycemic and insulin profile, and lipid profile (*p* ≤ 0.05). Similarly, 12 studies underlined significant effects in inflammation and immune response biomarkers, including TNF-α, IL-6, IL-10, C-reactive protein, leptin, and adiponectin (*p* ≤ 0.05). On the other hand, cardiac biomarkers were assessed in three studies without reporting significant differences after exercise therapy (p = NS). The quality assessment identified 19 RCTs as high-quality studies and three RCTs of low quality.

**Conclusion:** Our findings reveal significant biochemical perturbations in key molecules induced by physical exercise in patients with BC, suggesting room for the implementation of actionable biomarkers. Future research might clarify the role of biomarkers on treatment effectiveness monitoring, to optimize rehabilitative strategies tailored to patient’s needs.

## 1 Introduction

Breast cancer (BC) is the most common malignancy in women and one of the most common causes of cancer-related death worldwide (Sung et al., 2021). Due to the advances in early diagnosis and clinical management, the survival rate of these women is steadily increasing, paralleled by long-term disabling consequences, both cancer-related and treatment-related (D'Egidio et al., 2017; Nardin et al., 2020). The optimal management of BC survivors is a critical issue in the current literature with a growing number of reports that underlined the need for effective therapeutic strategies to improve the physical impairment and health-related quality of life (HR-QoL) of these women (Invernizzi et al., 2020c; Zhu et al., 2021).

In this scenario, rehabilitation plays a key role in the management of BC survivors with growing evidence highlighting its positive effects in improving functional outcomes and psychosocial well-being of BC patients (Reid-Arndt et al., 2009; Zaidi et al., 2017; Kudre et al., 2020; Sleight et al., 2022). Hence, rehabilitation might improve the symptoms of patients with breast cancer–related lymphedema (Invernizzi et al., 2019; Michelotti et al., 2019; de Sire et al., 2021a; Carretti et al., 2022; Muñoz-Alcaraz et al., 2022; Omar et al., 2022), cancer-related fatigue (Wirtz and Baumann, 2018; Adams et al., 2019; Invernizzi et al., 2020b; Marechal et al., 2020; Licht et al., 2021), axillary web syndrome (de Sire et al., 2020; Ravichandran et al., 2020; Tay et al., 2021), aromatase inhibitor–induced arthralgia (Harris et al., 2012; Winters-Stone et al., 2012; Grizzi et al., 2020), and cancer treatment–induced bone loss (Invernizzi et al., 2020a; de Sire et al., 2021b; Pagnotti et al., 2021; Singh and Toohey, 2022). In addition to these encouraging approaches, the rehabilitation management of these disabling sequelae is still a challenge, and several questions remain unanswered about the most effective and tailored interventions in real-world clinical practice (Invernizzi et al., 2020e).

In recent years, clinical biomarkers have been fully integrated into cancer management. In breast cancer, estrogen receptor (ER), progesterone receptor (PR), and human epidermal growth factor receptor 2 (HER2) are currently considered a milestone in the clinical decision-making process, providing crucial information about prognosis, and predicting response to cancer treatments (Gamble et al., 2021; Criscitiello et al., 2022).

Although BC management is biomarker-based, the treatment of BC sequelae is still challenging and no clear indications are currently available to precisely individualize exercise interventions (Invernizzi et al., 2020c; Ballinger et al., 2021). Moreover, recent research underlined that the clinical management of survivorship issues should include a framework of distinct interventions including physical therapies, rehabilitation counseling, dietary interventions, and exercise training, in order to expand the new concept of tailored cancer rehabilitation.

In this context, molecular biomarkers, previously understudied in rehabilitation, might become the cornerstone of a modern approach to cancer-related disability, promoting the implementation of a multidisciplinary approach to BC rehabilitation (Esteva et al., 2019; Invernizzi et al., 2020d). On the other hand, several reports emphasized the positive effects of physical exercise and rehabilitation in increasing anti-inflammatory cytokines concentrations and promoting the release of anti-inflammatory regulatory T-cells (Pierce et al., 2009; Mancuso, 2016). This hypothesis has been supported by preclinical and clinical studies highlighting the key role of physical exercise in the regulation of chemokine expression, promoting cytotoxic immune cell activity and downregulating suppressor immune cells (Upadhyay et al., 2018; Liu et al., 2020; Bartlett et al., 2021). Accordingly, it is widely accepted that chronic inflammation biomarkers (i.e., interleukin 6 (IL-6), tumor necrosis factor-α (TNF-α), macrophage migration inhibitory factor (MIF), and C-reactive protein (CRP)) might have a role in the oncogenesis process, promoting their oncogenic effects in both genetic and epigenetic alterations (Trinchieri, 2012; Zitvogel et al., 2017; Barabutis et al., 2018). In addition, other potential oncogenic mechanisms might be the target of the exercise-induced positive effects on cancer progression, including the modulation of metabolic homeostasis, hormone level regulation, improvement in immune surveillance, and the reduction of oxidative stress (Koelwyn et al., 2015).

Albeit rehabilitation is currently considered an effective non-pharmacological treatment to improve HR-QoL of BC women (Harris et al., 2012; Invernizzi et al., 2020b; Invernizzi et al., 2020c), evidence supporting precise monitoring of biological effects of rehabilitation interventions is lacking. Moreover, to the best of our knowledge, no previous systematic reviews summarized the currently available molecular biomarkers to assess the biological effects of different rehabilitative interventions in BC survivors.

The aim of this systematic review of randomized controlled trials (RCTs) was to provide a broad overview of potential molecular biomarkers that might guide clinicians and researchers to perform a more precise monitoring of biological effects of rehabilitation for BC women.

## 2 Methods

### 2.1 Registration

This systematic review of RCTs has been planned and performed following the Preferred Reporting Items for Systematic Reviews and Meta-Analyses (PRISMA) statement (Page et al., 2021). On 9th March 2022, a preliminary search of the PROSPERO register (https://www.crd.york.ac.uk/prospero) has been performed to assess systematic reviews or meta-analyses on the same topic without finding similar reviews already registered.

Therefore, the study protocol has been submitted to PROSPERO and accepted on 25 April 2022 (registration number CRD42022319908, available at https://www.crd.york.ac.uk/prospero/display_record.php?RecordID=319908).

### 2.2 Search strategy

Five databases on medical sciences and physical and rehabilitation medicine were systematically searched on 10th March 2022, without publication date restrictions. The databases are PubMed/Medline, Scopus, Cochrane Central Register of Controlled Trials (CENTRAL), Physiotherapy Evidence Database (PEDro), and Web of Science (WoS). Two investigators independently searched the databases. The search strategy is reported in detail in Supplementary Table S1.

### 2.3 Selection criteria

We considered as eligible the studies satisfying the following PICO model (Huang et al., 2006) criteria:

(1) P) Participants: adult women (aged 18 years and older) surgically treated for non-metastatic breast cancer.
(2) Intervention: rehabilitation treatment as exclusive intervention administered during or after cancer treatments. Rehabilitation treatment administered before cancer treatment has not been considered.
(3) C) Comparator: any comparator including pharmacological, non-pharmacological, or no treatment.
(4) O) Outcome: molecular biomarkers modifications assessed with blood samples or tissue biopsy. More in detail, it has been considered that molecular biomarkers assessing rehabilitation effects in terms of bioenergetic metabolism, immune system modulation, inflammation, and cardiovascular system.

Moreover, we considered for eligibility only the RCTs published in international peer-reviewed journals. The exclusion criteria are as follows: 1) studies involving animals; 2) language other than English; 3) pregnancy or clinical instability; 4) conference abstracts, masters, or doctorate theses.

### 2.4 Study screening and eligibility assessment

After duplication removal, two investigators independently reviewed the title and abstracts of retrieved articles to choose relevant articles. Any discordance was resolved by collegial discussion. If consensus was not achieved, a third reviewer was asked. All the reports that met the inclusion and exclusion criteria were screened in full text.

The full-text articles were screened by the same investigators and the records that met the eligibility criteria were included in the data extraction. Any disagreements between the two investigators were discussed with a third reviewer to reach a consensus.

### 2.5 Data extraction and synthesis

All data were assessed and extracted independently from full-text documents into Excel by two authors. Any disagreement between the two reviewers was solved by collegial discussion among the authors. In case of disagreement, a third author was asked.

The following data are extracted: 1) title, 2) authors, 3) publication year, 4) nationality, 5) participants’ characteristics (number, mean age and age range, and BMI), 6) tumor characteristics, 7) interventions’ characteristics (type of rehabilitative treatment, number of sessions, intensity, and duration of intervention), 8) comparator, 9) outcomes, and 10) main findings.

All the data extracted are summarized in tables. Subgroup analysis will be performed based on rehabilitative intervention administered and based on biomarkers assessed.

### 2.6 Quality assessment and risk of bias

The quality of the studies included was assessed independently by two of the authors, in accordance with the Jadad scale (Jadad et al., 1996). In case of discordances, it was solved by discussion between the authors or by asking a third reviewer. The items assessed are as follows: 1) random sequence generation, 2) appropriate randomization, 3) blinding of participants or personnel, 4) blinding of outcome assessors, and 5) withdrawals and dropouts. A Jadad score between 3 and 5 points was considered high quality.

The Cochrane risk-of-bias tool for randomized trials (RoBv.2) (Sterne et al., 2019) was used for risk of bias assessment. The domains assessed by RoBv.2 are: 1) random sequence generation, 2) allocation concealment, 3) blinding of participants and personnel, 4) blinding of outcome assessment, 5) incomplete outcome data, 6) selective outcome reporting, and 7) other bias. According to these items, bias was classified as low, high, or unclear.

## 3 Results

### 3.1 Study characteristics

**FIGURE 1:**
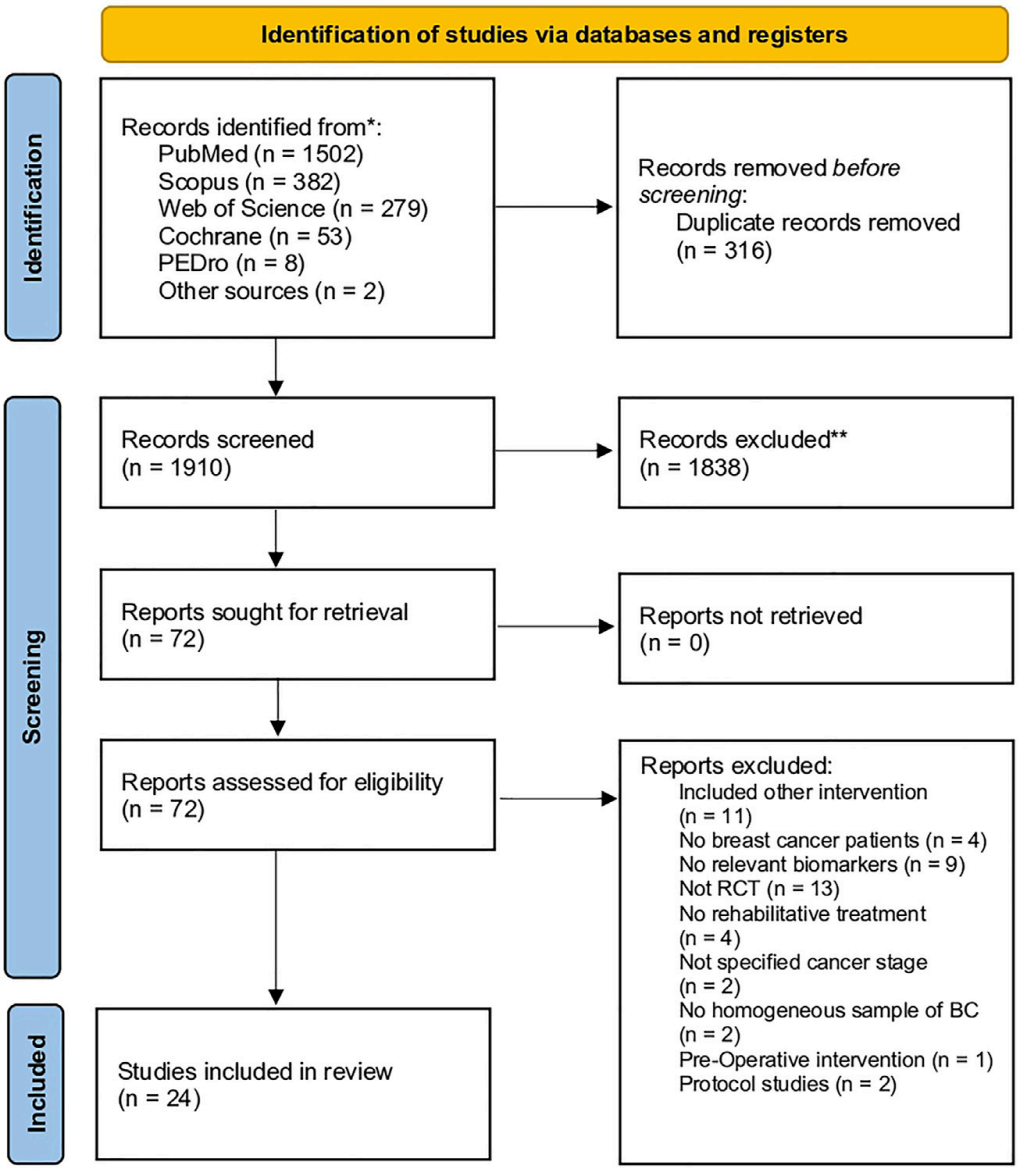
PRISMA 2020 flow chart.

Through our search strategy, 2,225 records were identified from the five databases, while two studies have been identified from other sources. Figure 1 shows the PRISMA 2020 flow diagram of the search process in detail. After duplication removal, 1,910 studies were assessed for eligibility and screened for the title and abstract. After the exclusion of 1,838 records, 72 full-text records were assessed for eligibility. Forty-eight articles were excluded for inconsistency with the eligibility criteria (13 were not RCT, two were protocol studies, four did not include BC patients, two did not assess a homogeneous sample of BC patients, two did not specify the cancer stage, 11 included other interventions, four did not perform any rehabilitative treatment, nine did not evaluate biomarkers, and one performed pre-operative treatment). The studies assessed in full text and the reasons for exclusions are presented in detail in Figure 1 and Supplementary Table S2. As a result, 24 studies were included in the present work (Fairey et al., 2003; Schmitz et al., 2005; Ligibel et al., 2008; Irwin et al., 2009; Dolan et al., 2010; Janelsins et al., 2011; Sprod et al., 2012; Guinan et al., 2013; Jones et al., 2013; Lahart et al., 2016; Kim et al., 2017; Dieli-Conwright et al., 2018a; Dieli-Conwright et al., 2018b; de Paulo et al., 2018; Kirkham et al., 2018; Mijwel et al., 2018; Alizadeh et al., 2019; Hartman et al., 2019; Lee et al., 2019; Lee et al., 2020a; Chang et al., 2020; Pagola et al., 2020; Ansund et al., 2021; Hiensch et al., 2021).

### 3.2 Study characteristics

The RCTs included were published between 2003 (Fairey et al., 2003) and 2021 (Ansund et al., 2021). The nationalities of the studies included in this review are as follows: 11 studies (45.8%) were conducted in the United States (Schmitz et al., 2005; Ligibel et al., 2008; Irwin et al., 2009; Janelsins et al., 2011; Sprod et al., 2012; Jones et al., 2013; Dieli-Conwright et al., 2018a; Dieli-Conwright et al., 2018b; Hartman et al., 2019; Lee et al., 2019; Lee et al., 2020a), three (12.5%) were conducted in Canada (Fairey et al., 2003; Dolan et al., 2010; Kirkham et al., 2018), one (4.1%) was conducted in Brazil, five studies (20.8%) were European (three conducted in Sweden (Ansund et al., 2021; Hiensch et al., 2021), one in Ireland (Guinan et al., 2013), one in the United Kingdom (Lahart et al., 2016), and one in Spain (Pagola et al., 2020)), and three studies (12.5%) were Asian (two conducted in South Korea (Kim et al., 2017; Chang et al., 2020) and one in Iran (Alizadeh et al., 2019)). All the characteristics of the included studies are shown in detail in Supplementary Table S3.

### 3.3 Study participants

In the present systematic review, 1,479 subjects were assessed in the included studies, all females (100%). More in detail, 816 BC patients were included in the intervention groups, while 654 BC patients were included in the control groups. The ages of the subjects ranged from 45.05 ± 9.04 years (Guinan et al., 2013) to 66.6 ± 9.6 years (de Paulo et al., 2018). The body composition was assessed by BMI and it ranged from 22.7 ± 2.6 kg/m^2^ (Chang et al., 2020) to 33.5 ± 5.5 kg/m^2^ (Lee et al., 2019). However, it should be noted that one study (Lahart et al., 2016) reported the number of patients per range of BMI (<25, 25–29.9, and ≥30 kg/m^2^), while two studies (Guinan et al., 2013; Mijwel et al., 2018) did not report the BMI of the study participants.

The stages of BC patients ranged from 0 (Irwin et al., 2009; Janelsins et al., 2011; Sprod et al., 2012; Jones et al., 2013) to IIIB (Fairey et al., 2003; Janelsins et al., 2011; Sprod et al., 2012), while the cancer treatments received included surgical interventions, chemotherapy, radiotherapy, and hormonal therapy (Fairey et al., 2003; Schmitz et al., 2005; Ligibel et al., 2008; Irwin et al., 2009; Dolan et al., 2010; Janelsins et al., 2011; Sprod et al., 2012; Guinan et al., 2013; Jones et al., 2013; Lahart et al., 2016; Kim et al., 2017; Dieli-Conwright et al., 2018a; Dieli-Conwright et al., 2018b; de Paulo et al., 2018; Kirkham et al., 2018; Alizadeh et al., 2019; Hartman et al., 2019; Lee et al., 2019; Chang et al., 2020; Pagola et al., 2020; Ansund et al., 2021; Hiensch et al., 2021). Supplementary Table S3 shows further details on the cancer stage and cancer treatments received in each study included.

Control groups were composed of BC patients that underwent active control, usual care, no intervention, or psychosocial support therapy. In particular, 11 studies (Fairey et al., 2003; Irwin et al., 2009; Dolan et al., 2010; Sprod et al., 2012; Jones et al., 2013; Dieli-Conwright et al., 2018a; Dieli-Conwright et al., 2018b; Mijwel et al., 2018; Alizadeh et al., 2019; Lee et al., 2020a; Hiensch et al., 2021) assessed rehabilitation treatment compared with usual care, nine studies did not report other interventions (Schmitz et al., 2005; Ligibel et al., 2008; Guinan et al., 2013; Lahart et al., 2016; Kim et al., 2017; Kirkham et al., 2018; Hartman et al., 2019; Lee et al., 2019; Chang et al., 2020), and one study assessed rehabilitation treatment compared with psychosocial support therapy (Janelsins et al., 2011). Lastly, three studies assessed an active control including physical exercises according to 2010 guidelines of the American College of Sports Medicine (Ansund et al., 2021), stretching and relaxation program (de Paulo et al., 2018), and physical training with lower intensity compared to the intervention group (Pagola et al., 2020).

Control groups and the treatments received are characterized in detail in Supplementary Table S3.

### 3.4 Rehabilitation approaches

Out of the 22 studies assessed, high heterogeneity of rehabilitative treatments was proposed in the studies included. High-intensity interval training (HIIT) was assessed in five studies (Mijwel et al., 2018; Alizadeh et al., 2019; Lee et al., 2020a; Ansund et al., 2021; Hiensch et al., 2021), exploring the effects of different training protocols. The study from Alizadeh et al. (2019) assessed a HIIT protocol on a motorized treadmill, while Lee et al. (2020a) assessed a HIIT protocol on a stationary bicycle.

Interestingly, Mijwel et al. (2018), Ansund et al. (2021), and Hiensch et al. (2021) assessed the effects of HIIT combined with resistance exercise training (RET) or aerobic exercise training (AET).

In contrast, AET alone was assessed in six studies (Fairey et al., 2003; Irwin et al., 2009; Guinan et al., 2013; Jones et al., 2013; Kirkham et al., 2018; Hartman et al., 2019), while RET was assessed in one study (Schmitz et al., 2005). Interestingly, Dolan et al. (2010) assessed the effects of RET and AET in two different arms of treatment, compared to usual care.

On the other hand, combined exercise training (CET) is the most studied training modality, with eight studies assessing different CET programs (Ligibel et al., 2008; Kim et al., 2017; Dieli-Conwright et al., 2018a; Dieli-Conwright et al., 2018b; de Paulo et al., 2018; Lee et al., 2019; Chang et al., 2020; Pagola et al., 2020). Lastly, Sprod et al. (2012) and Janelsins et al. (2011) assessed a Tai Chi Chuan exercise intervention. Just one study did not report exercise modality (Lahart et al., 2016).

Interestingly, the rehabilitative treatments were supervised in 23 studies. On the other hand, five studies (Ligibel et al., 2008; Irwin et al., 2009; Guinan et al., 2013; Jones et al., 2013; Hiensch et al., 2021) assessed also a home-based intervention and one study (Schmitz et al., 2005) a non-supervised phase. Lastly, the RCT by Lahart et al. (2016) assessed home-based rehabilitation only.

The exercise protocols were assessed after chemotherapy and/or radiotherapy treatments in the majority of the studies included (*n* = 18). Supplementary Table S3 shows in detail the interventions’ characteristics of the rehabilitation treatments assessed in the present review.

### 3.5 Biomarker modifications—Inflammation biomarkers

Our systematic review included 12 RCTs (Janelsins et al., 2011; Sprod et al., 2012; Guinan et al., 2013; Jones et al., 2013; Kim et al., 2017; Dieli-Conwright et al., 2018a; Dieli-Conwright et al., 2018b; de Paulo et al., 2018; Alizadeh et al., 2019; Hartman et al., 2019; Pagola et al., 2020; Hiensch et al., 2021) assessing inflammation biomarkers that might have a role in monitoring the biological effect of rehabilitation. More in detail, the following biomarkers were assessed in blood samples:

- C-reactive protein (CRP) is one of the most studied biomarkers in BC patients receiving physical activity intervention. In particular, seven studies (Dolan et al., 2010; Guinan et al., 2013; Jones et al., 2013; Kim et al., 2017; Dieli-Conwright et al., 2018a; Dieli-Conwright et al., 2018b; Hartman et al., 2019) assessed CRP changes after rehabilitation; however, only three RCTs (Dolan et al., 2010; Dieli-Conwright et al., 2018a; Dieli-Conwright et al., 2018b) reported significant changes (*p* ≤ 0.05) after treatment. Interestingly, all the studies reporting significant results in terms of CRP blood level modifications assessed the effects of a CET protocol.
- Interleukin (IL)-6 was assessed in seven studies (Janelsins et al., 2011; Sprod et al., 2012; Jones et al., 2013; Dieli-Conwright et al., 2018a; Dieli-Conwright et al., 2018b; Alizadeh et al., 2019; Hiensch et al., 2021), while significant improvement (*p* ≤ 0.05) were reported in four RCTs (Dieli-Conwright et al., 2018a; Dieli-Conwright et al., 2018b; Alizadeh et al., 2019; Hiensch et al., 2021). Significant results in IL-6 blood levels were reported after HIIT (Alizadeh et al., 2019; Hiensch et al., 2021) or CET interventions (Dieli-Conwright et al., 2018a; Dieli-Conwright et al., 2018b).
- Leptin was assessed in three studies reporting significant improvement (*p* ≤ 0.05) after the exercise intervention (Kim et al., 2017; Dieli-Conwright et al., 2018a; Dieli-Conwright et al., 2018b). Interestingly, all the studies considered assessed CET protocols (Kim et al., 2017; Dieli-Conwright et al., 2018a; Dieli-Conwright et al., 2018b).
- Tumor necrosis factor (TNF)-*α* was assessed in three studies (Jones et al., 2013; Dieli-Conwright et al., 2018a; Alizadeh et al., 2019), while two studies (Dieli-Conwright et al., 2018a; Alizadeh et al., 2019) underlined significant reduction (*p* ≤ 0.05) after HIIT (Alizadeh et al., 2019) or after CET interventions (Dieli-Conwright et al., 2018a).
- Adiponectin was assessed in three RCTs (Kim et al., 2017; Dieli-Conwright et al., 2018a; Dieli-Conwright et al., 2018b), while two studies (Dieli-Conwright et al., 2018a; Dieli-Conwright et al., 2018b) reported a significant increase in adiponectin blood levels (*p* ≤ 0.05) after CET.
- IL-8 was assessed in two studies (Dieli-Conwright et al., 2018a; Dieli-Conwright et al., 2018b), underlining a significant reduction (*p* ≤ 0.05) after two different CET protocols.
- IL-10 was assessed in two studies (Alizadeh et al., 2019; Hiensch et al., 2021), highlighting significant improvement (*p* ≤ 0.05) after HIIT on a treadmill (Alizadeh et al., 2019), and after AET combined with HIIT (Hiensch et al., 2021).
- Interferon (INF)-*γ* was assessed in two studies (Janelsins et al., 2011; Alizadeh et al., 2019); however, no significant results in terms of interferon (INF)-γ blood levels were reported.
- C-X-C motif chemokine (CXCL) 9 was assessed in one study (Hiensch et al., 2021), which showed a significant increase (*p* ≤ 0.05) after HIIT combined with AET or RET.
- CD40-L was assessed in one study (Hiensch et al., 2021), reporting a significant reduction (*p* ≤ 0.05) after RET combined with HIIT.
- Epidermal growth factor (EGF) was assessed in one study (Hiensch et al., 2021) underlining significant changes (*p* ≤ 0.05) after RET combined with HIIT.
- CASP-8 was assessed in one study (Hiensch et al., 2021), which showed a significant reduction (*p* ≤ 0.05) after RET combined with HIIT.
- Fas antigen ligand (FasL) was assessed in one study (Hiensch et al., 2021), which showed a significant increase (*p* ≤ 0.05) after AET combined with HIIT.
- IL-1β was assessed in one study (Alizadeh et al., 2019), without reporting significant improvement after HIIT on a treadmill.
- IL-2 was assessed in one RCT (Janelsins et al., 2011); however, no significant differences were reported after a Tai Chi Chuan exercise protocol.
- IL-4 was assessed in one study (Alizadeh et al., 2019), which showed significant improvement (*p* ≤ 0.05) after HIIT on a treadmill.
- IL-6/IL-10 ratio was assessed in one study (Alizadeh et al., 2019), which showed significant improvement (*p* ≤ 0.05) after HIIT on a treadmill.
- Neutrophil-to-lymphocyte ratio was assessed in one study (Pagola et al., 2020), which showed a significant decrease after high-intensity training (*p* ≤ 0.05).
- Cortisol was assessed in one study (Sprod et al., 2012); however, no significant results were reported after the exercise intervention.
- Heat shock protein (HSP) 70 was assessed in one study (Alizadeh et al., 2019), which showed significant improvement (*p* ≤ 0.05) after HIIT.
**-** Decorin (DCN), CCL17, ICOS ligand (ICOSLG), MHC class I polypeptide-related sequence A/B (MIC A/B), macrophage colony–stimulating factor (CSF)-1, TNF-related apoptosis-inducing ligand (TRAIL) were assessed only in the RCT by Hiensch et al. (2021); however, no significant results were reported after a HIIT protocol combined with RET or AET.

One study (Dieli-Conwright et al., 2018b) assessed the following inflammation biomarkers in adipose tissue biopsy:

- M1 pro-inflammatory (macrophage subtype), M2 anti-inflammatory (macrophage subtype), and adiponectin (cytokine secretions in adipose tissue) showed significant changes (*p* ≤ 0.05) after a CET protocol (Dieli-Conwright et al., 2018b).
- IL-12 p40 and IL-12 p70 (cytokine secretions in adipose tissue) did not report significant changes (Dieli-Conwright et al., 2018b).

**FIGURE 2:**
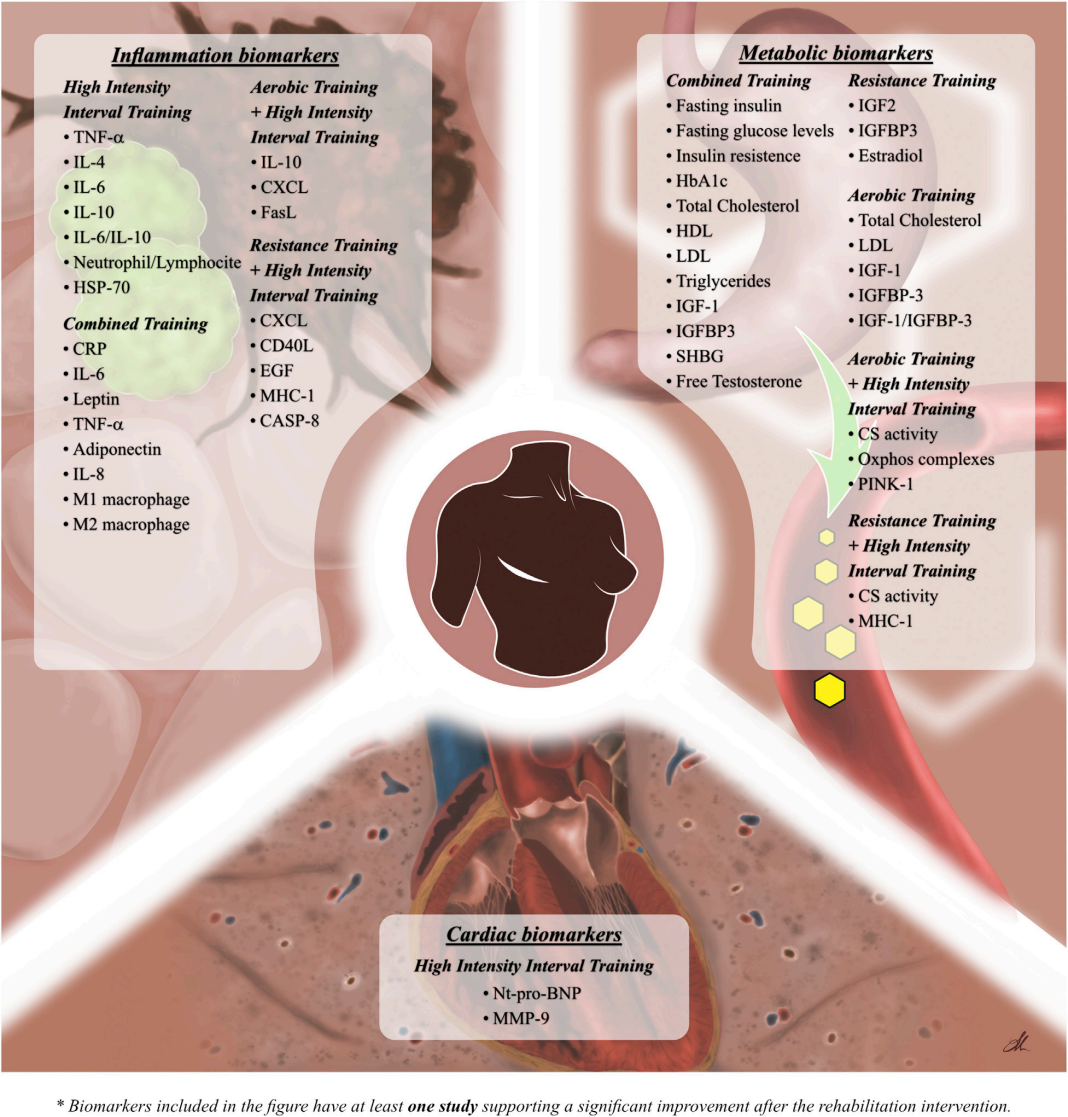
Figure shows the significant effects of the different training modality in molecular biomarkers modifications.

Figure 2 underlines inflammation biomarkers that significantly change after rehabilitation intervention.

### 3.6 Biomarker modifications—Metabolism biomarkers

Out of 24 studies, 16 studies (Fairey et al., 2003; Schmitz et al., 2005; Ligibel et al., 2008; Irwin et al., 2009; Janelsins et al., 2011; Sprod et al., 2012; Guinan et al., 2013; Lahart et al., 2016; Kim et al., 2017; Dieli-Conwright et al., 2018a; Dieli-Conwright et al., 2018b; de Paulo et al., 2018; Mijwel et al., 2018; Hartman et al., 2019; Lee et al., 2019; Chang et al., 2020) assessed the effects of rehabilitation in metabolism biomarkers. The following biomarkers were assessed in blood samples:

- Fasting insulin was the most studied metabolism biomarker, and it was assessed in 12 RCTs (Fairey et al., 2003; Schmitz et al., 2005; Ligibel et al., 2008; Irwin et al., 2009; Janelsins et al., 2011; Sprod et al., 2012; Guinan et al., 2013; Lahart et al., 2016; Kim et al., 2017; Dieli-Conwright et al., 2018a; Dieli-Conwright et al., 2018b; Chang et al., 2020). Interestingly, five studies (Ligibel et al., 2008; Kim et al., 2017; Dieli-Conwright et al., 2018a; Dieli-Conwright et al., 2018b; Chang et al., 2020) reported a significant decrease (*p* ≤ 0.05) in fasting insulin blood levels after different CET interventions.
- Fasting glucose blood levels were assessed in 10 studies (Fairey et al., 2003; Schmitz et al., 2005; Ligibel et al., 2008; Sprod et al., 2012; Guinan et al., 2013; Lahart et al., 2016; Dieli-Conwright et al., 2018a; Dieli-Conwright et al., 2018b; de Paulo et al., 2018; Chang et al., 2020). On the other hand, only two RCTs (Dieli-Conwright et al., 2018a; Dieli-Conwright et al., 2018b) reported a significant decrease (*p* ≤ 0.05) after the exercise intervention, consisting of two different CET protocols.
- Insulin resistance was assessed in nine RCTs (Fairey et al., 2003; Schmitz et al., 2005; Ligibel et al., 2008; Guinan et al., 2013; Lahart et al., 2016; Dieli-Conwright et al., 2018a; Dieli-Conwright et al., 2018b; Hartman et al., 2019; Chang et al., 2020) through the homeostatic model assessment index—insulin resistance (HOMA-IR, HOMA1-IR, and HOMA2-IR); four studies (Ligibel et al., 2008; Dieli-Conwright et al., 2018a; Dieli-Conwright et al., 2018b; Chang et al., 2020) found a significant improvement (*p* ≤ 0.05) after CET intervention;
- Glycosylated hemoglobin (HbA1c) was assessed in two studies (Guinan et al., 2013; Dieli-Conwright et al., 2018b), while significant results (*p* ≤ 0.05) were found in the RCTs by Dieli-Conwright et al. (2018b) after CET.
- Total cholesterol was assessed in six studies (Guinan et al., 2013; Lahart et al., 2016; Dieli-Conwright et al., 2018a; Dieli-Conwright et al., 2018b; de Paulo et al., 2018; Chang et al., 2020), reporting significant changes (*p* ≤ 0.05) in four RCTs after CET (Dieli-Conwright et al., 2018a; Dieli-Conwright et al., 2018b; Chang et al., 2020) or AET (Lahart et al., 2016) protocols.
- High density lipoprotein (HDL) was assessed in six studies (Guinan et al., 2013; Lahart et al., 2016; Dieli-Conwright et al., 2018a; Dieli-Conwright et al., 2018b; de Paulo et al., 2018; Lee et al., 2019), while three RCTs (Dieli-Conwright et al., 2018a; Dieli-Conwright et al., 2018b; Lee et al., 2019) underlined increases (*p* ≤ 0.05) after CET protocols.
- Low density lipoprotein (LDL) was assessed in six studies (Guinan et al., 2013; Lahart et al., 2016; Dieli-Conwright et al., 2018b; de Paulo et al., 2018; Lee et al., 2019; Chang et al., 2020), with a significant decrease (*p* ≤ 0.05) after three CET protocols (Dieli-Conwright et al., 2018b; Lee et al., 2019; Chang et al., 2020) or one AET protocol (Lahart et al., 2016).
- Triglycerides were assessed in six studies (Guinan et al., 2013; Lahart et al., 2016; Dieli-Conwright et al., 2018a; Dieli-Conwright et al., 2018b; de Paulo et al., 2018; Chang et al., 2020), only two RCTs (Dieli-Conwright et al., 2018a; Dieli-Conwright et al., 2018b) reported a significant decrease (*p* ≤ 0.05) after the intervention consisting of CET.
- Total cholesterol/HDL ratio was assessed in one study (Guinan et al., 2013), without reporting significant results.
- Insulin growth factor (IGF)-I was assessed in five studies (Schmitz et al., 2005; Irwin et al., 2009; Janelsins et al., 2011; Sprod et al., 2012; Dieli-Conwright et al., 2018a), showing significant results (*p* ≤ 0.05) in two studies. More in detail, one RCT reported a significant reduction (*p* ≤ 0.05) in IGF-I after CET (Dieli-Conwright et al., 2018a), while one RCT reported a significant reduction (*p* ≤ 0.05) after AET (Irwin et al., 2009).
- IGF-II was assessed in two studies (Fairey et al., 2003; Schmitz et al., 2005); however, just one study (Schmitz et al., 2005) reported a significant reduction (*p* ≤ 0.05) in IGF-II blood levels after RET.
- Insulin growth factor binding protein (IGFBP)-1 was assessed in four studies (Fairey et al., 2003; Schmitz et al., 2005; Janelsins et al., 2011; Sprod et al., 2012); however, all the studies assessed failed to demonstrate significant effects of physical exercise on IGFBP-1.
- IGFBP-2 was assessed in one study (Schmitz et al., 2005), without reporting significant effects.
- IGFBP-3 was assessed in six studies (Fairey et al., 2003; Schmitz et al., 2005; Irwin et al., 2009; Janelsins et al., 2011; Sprod et al., 2012; Dieli-Conwright et al., 2018a). Interestingly, three RCTs found a significant increase (*p* ≤ 0.05) after CET (Dieli-Conwright et al., 2018a), AET (Fairey et al., 2003), or RET (Schmitz et al., 2005) protocols. In contrast, one study (Irwin et al., 2009) reported a significant decrease (*p* ≤ 0.05) after AET intervention.
- IGF-I/IGFBP-3 molar ratio was assessed in one study (Fairey et al., 2003), which showed a significant decrease (*p* ≤ 0.05) after AET intervention.
- Sex hormone–binding globulin (SHBG) was assessed in one study (Dieli-Conwright et al., 2018a), reporting a significant increase (*p* ≤ 0.05) after CET intervention.
- Estradiol was assessed in one RCT (Dieli-Conwright et al., 2018a), which showed significant changes (*p* ≤ 0.05) after CET intervention.
- Free testosterone was assessed in one study (Dieli-Conwright et al., 2018a), underlining significant differences between groups after CET (*p* ≤ 0.05).

One study (Mijwel et al., 2018) assessed the following metabolism biomarkers in tissue biopsy:

- CS activity showed a significant decrease in CG (*p* ≤ 0.05). Moreover, significant differences between groups were reported in both the AET-HIIT group (*p* = 0.005) and RET-HIIT group (*p* = 0.027) compared to CG.
- Oxphos complex showed significant changes in the AT-HIIT group in terms of complex IV (*p* = 0.04). Moreover, significant differences were reported between the AT-HIIT group and CG in complex I (*p* = 0.003), complex II (*p* = 0.007), and complex IV (*p* = 0.004), while significant differences were found between AT-HIIT and RT-HIIT in complex I (*p* = 0.011), complex II (*p* = 0.005), and complex IV (*p* = 0.002).
- MHC isoforms showed a significant decrease in MHC isoform type I in CG (*p* = 0.006). There were significant differences in MHC isoform type I between RT-HIIT and CG (*p* = 0.016).
- PINK1 showed significant within-group differences in CG (*p* = 0.031). Moreover, there were significant differences in PINK1 between AT-HIIT and CG (*p* = 0.012).
- SOD2 showed significant changes in CG (*p* = 0.005). No significant changes were reported in both AET-HIIT and RET-HIIT groups.

Metabolic biomarkers showing significant changes after rehabilitation intervention are summarized in Figure 2.

### 3.7 Biomarker modification—Cardiac biomarkers

Few studies assessed cardiac biomarkers in BC patients undergoing chemotherapy. More in detail, four RCTs (Dolan et al., 2010; Kirkham et al., 2018; Lee et al., 2020a; Ansund et al., 2021) assessed the following biomarkers in blood samples:

- Cardiac troponin T (cTnT) was assessed in two studies (Kirkham et al., 2018; Ansund et al., 2021); however, no significant differences were reported in terms of cTnT in patients undergoing a rehabilitation intervention.
- N-terminal prohormone of the brain natriuretic peptide (Nt-pro-BNP) was assessed in two RCTs (Kirkham et al., 2018; Ansund et al., 2021). Interestingly, significant differences between the groups (*p* ≤ 0.05) were reported at 1-year follow-up when the mean level of Nt-pro-BNP was significantly higher in the control group than that in the intervention groups (*p* = 0.036) (Ansund et al., 2021).
**-** Hemoglobin (Hb) was assessed in two studies (Dolan et al., 2010; Kirkham et al., 2018) without reporting significant differences between groups after the exercise intervention.
- Matrix metalloproteinases (MMP) 1 was assessed in one study (Lee et al., 2020a). No significant differences were reported in both within-group analysis and between-group analysis.
- MMP-2 was assessed in one study (Lee et al., 2020a). Significant changes were reported in both the intervention group (*p* = 0.007) and control group (*p* = 0.003), without reporting significant differences between groups.
- MMP-7 was assessed in one study (Lee et al., 2020a). No significant changes were reported in both groups. Moreover, no significant differences were found between groups.
- MMP-9 was assessed in one RCT (Lee et al., 2020a). Significant differences were reported in the intervention group after a HIIT protocol (*p* = 0.01). However, no significant differences between groups were found.
- MMP-10 was assessed in one study (Lee et al., 2020a). No significant differences were reported in both within-group analysis and between-group analysis.
- Tissue inhibitor of matrix metalloproteinases (TIMP) 1 was assessed in one study (Lee et al., 2020a). There were no significant differences after a HIIT treatment in both groups.
- TIMP-2 was assessed in one RCT (Lee et al., 2020a). No significant changes were reported in both groups. Moreover, no significant differences were found between groups.

Significant modifications in cardiac biomarkers after rehabilitation intervention are shown in Figure 2.

### 3.8 Quality assessment and risk of bias

#### 3.8.1 Quality assessment and risk of bias

According to the Jadad scale (Jadad et al., 1996), studies with a score from 3 to 5 were considered of high quality, while a lower score was considered of low quality. Twenty-one (87.5%) of the RCTs included (Fairey et al., 2003; Schmitz et al., 2005; Ligibel et al., 2008; Irwin et al., 2009; Janelsins et al., 2011; Sprod et al., 2012; Guinan et al., 2013; Jones et al., 2013; Lahart et al., 2016; Kim et al., 2017; Dieli-Conwright et al., 2018a; Dieli-Conwright et al., 2018b; Kirkham et al., 2018; Mijwel et al., 2018; Alizadeh et al., 2019; Lee et al., 2019; Lee et al., 2020b; Chang et al., 2020; Pagola et al., 2020; Ansund et al., 2021; Hiensch et al., 2021) resulted in high-quality studies. Lower quality was found in three (12.5%) of the studies (Dolan et al., 2010; de Paulo et al., 2018; Hartman et al., 2019). It should be noted that due to the intrinsic nature of the rehabilitative intervention, it is impossible to blind operators and participants, which resulted in all studies (*n* = 24, 100%) (Fairey et al., 2003; Schmitz et al., 2005; Ligibel et al., 2008; Irwin et al., 2009; Dolan et al., 2010; Janelsins et al., 2011; Sprod et al., 2012; Guinan et al., 2013; Jones et al., 2013; Lahart et al., 2016; Kim et al., 2017; Dieli-Conwright et al., 2018a; Dieli-Conwright et al., 2018b; de Paulo et al., 2018; Kirkham et al., 2018; Mijwel et al., 2018; Alizadeh et al., 2019; Hartman et al., 2019; Lee et al., 2019; Lee et al., 2020b; Chang et al., 2020; Pagola et al., 2020; Ansund et al., 2021; Hiensch et al., 2021) scoring 0 to the related item; thus, if we adjust the score according to this limitation, all studies might be considered of high quality. Table 1 shows, in detail, the score of each sub-item of the Jadad scale for the RCTs included.

**TABLE 1:** Quality assessment of the studies included in the present systematic review.

Article	Domain					Score
	Random sequence generation	Appropriate randomization	Blinding of participant or personnel	Blinding of outcome assessor	Withdrawal and dropout	
Alizadeh et al. (2019)	1	1	0	1	1	4
Ansund et al. (2021)	1	1	0	0	1	3
Chang et al. (2020)	1	1	0	1	1	4
de Paulo et al. (2018)	1	0	0	0	1	2
Dieli-Conwright et al., 2018	1	1	0	0	1	3
Dieli-Conwright et al., 2018 bis	1	1	0	1	1	4
Dolan et al. (2010)	1	1	0	0	0	2
Fairey et al. (2003)	1	1	0	0	1	3
Guinan et al. (2013)	1	1	0	1	1	4
Hartman et al. (2019)	1	1	0	0	0	2
Hiensch et al. (2021)	1	1	0	1	1	4
Irwin et al. (2009)	1	1	0	1	1	4
Janelsins et al. (2011)	1	1	0	0	1	3
Jones et al. (2013)	1	1	0	1	1	4
Kim et al. (2017)	1	1	0	1	1	4
Kirkham et al., 2017	1	1	0	1	1	4
Lahart et al. (2016)	1	1	0	0	1	3
Lee et al. (2019)	1	1	0	0	1	3
Lee et al. (2020b)	1	1	0	0	1	3
Ligibel et al. (2008)	1	1	0	1	1	4
Mijwel et al. (2018)	1	1	0	1	1	4
Pagola et al. (2020)	1	1	0	1	1	4
Schmitz et al. (2005)	1	1	0	1	1	4
Sprod et al. (2012)	1	1	0	0	1	3

Points are awarded as follows: study described as randomized, 1 point; appropriate randomization, 1 point; subjects blinded to intervention, 1 point; evaluator blinded to intervention, 1 point; description of withdrawals and dropouts, 1 point.

**FIGURE 3:**
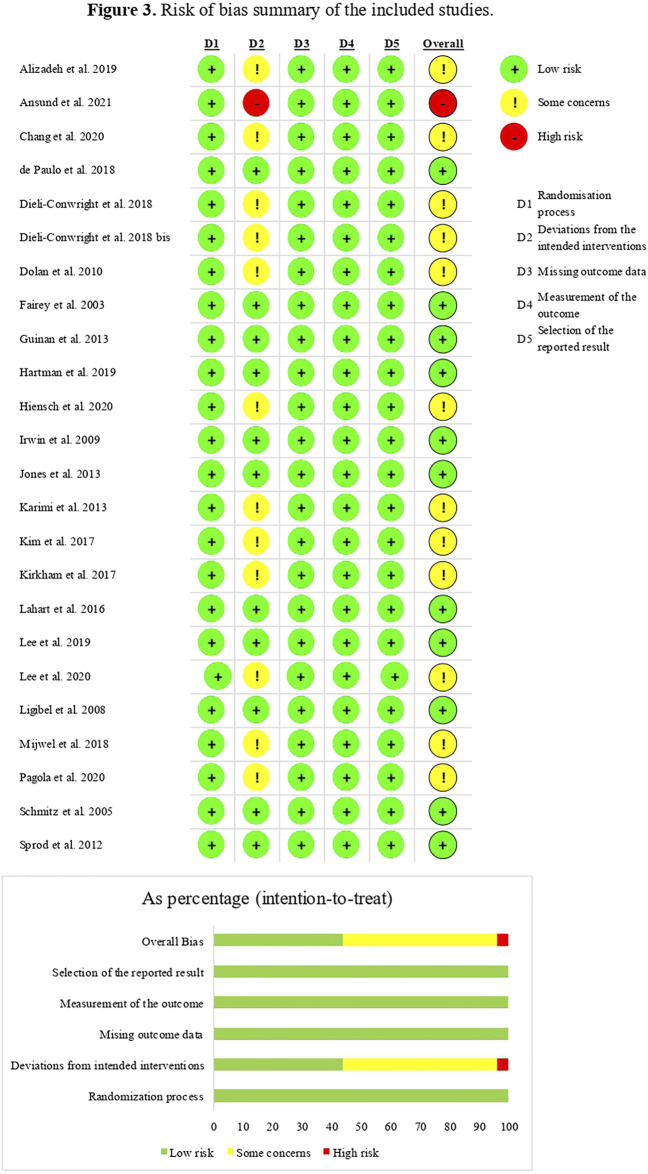
Risk of bias of the included studies according to the RoB2.

The risk of bias was assessed by RoBv.2 (Sterne et al., 2019). The process showed that all studies (*n* = 24, 100%) (Fairey et al., 2003; Schmitz et al., 2005; Ligibel et al., 2008; Irwin et al., 2009; Dolan et al., 2010; Janelsins et al., 2011; Sprod et al., 2012; Guinan et al., 2013; Jones et al., 2013; Lahart et al., 2016; Kim et al., 2017; Dieli-Conwright et al., 2018a; Dieli-Conwright et al., 2018b; de Paulo et al., 2018; Kirkham et al., 2018; Mijwel et al., 2018; Alizadeh et al., 2019; Hartman et al., 2019; Lee et al., 2019; Lee et al., 2020b; Chang et al., 2020; Pagola et al., 2020; Ansund et al., 2021; Hiensch et al., 2021) ensured low risk of bias for the randomization process, missing outcome data, measurement of the outcome, and selection of the reported result. The major concerns were regarding possible deviations from the intended interventions; this was mainly due to the lack of an appropriate analysis estimating the effect of assignment to intervention. In particular, 12 studies (50%) (Dolan et al., 2010; Janelsins et al., 2011; Kim et al., 2017; Dieli-Conwright et al., 2018a; Dieli-Conwright et al., 2018b; Kirkham et al., 2018; Mijwel et al., 2018; Alizadeh et al., 2019; Lee et al., 2020b; Chang et al., 2020; Pagola et al., 2020; Hiensch et al., 2021) showed some concerns in the second domain because it was not mentioned in the implementation of the intention-to-treat analysis; this evaluation leads to an overall presence of concerns regarding the risk of bias of the studies. One study (4.2%) (Ansund et al., 2021) showed the high risk of bias due to the utilization of per-protocol analysis, which might increase the risk of bias, resulting in an overall high concern score. Further details are shown in Figure 3.

## 4 Discussion

Rehabilitation is an important treatment approach for the long-term management of BC survivors, with growing evidence supporting its effectiveness in improving physical function, psychosocial well-being, and HR-QoL in these women (Invernizzi et al., 2020c). Although exercise-induced benefits in cancer patients are widely documented, to date, evidence about molecular biomarkers to monitor the biological therapeutic effects of physical exercise is lacking. On the other hand, recent research emphasized the need to integrate innovative approaches supported by specific biomarkers in cancer treatments (Invernizzi et al., 2020d; Fusco et al., 2020), and rehabilitation in BC survivors should not be overlooked.

In light of these considerations, this systematic review of RCTs provided a broad overview of currently available biomarkers that might be influenced by physical exercise, characterizing the exercise modalities and summarizing the evidence supporting biomarker implementation in the clinical setting in order to guide physicians in a precise prescription of individualized rehabilitation plans.

Interestingly, our data showed that physical exercise significantly decreased pro-inflammatory biomarkers in 12 studies (Janelsins et al., 2011; Sprod et al., 2012; Guinan et al., 2013; Jones et al., 2013; Kim et al., 2017; Dieli-Conwright et al., 2018a; Dieli-Conwright et al., 2018b; de Paulo et al., 2018; Alizadeh et al., 2019; Hartman et al., 2019; Pagola et al., 2020; Hiensch et al., 2021). In contrast, several reports failed to underline the significant effects of specific inflammation biomarkers (Fairey et al., 2003; Schmitz et al., 2005; Ligibel et al., 2008; Irwin et al., 2009; Dolan et al., 2010; Lahart et al., 2016; Kirkham et al., 2018; Lee et al., 2019; Chang et al., 2020; Ansund et al., 2021). These conflicting data suggested that the positive effects in terms of exercise-induced immune regulation might be strictly related to exercise characteristics.

In accordance with our data, the previous systematic review by Abbasi et al. (2022) reported a significant reduction in CRP levels following the physical exercise in BC survivors; however, the authors failed to find significant changes in other relevant inflammation biomarkers, including TNF-α, IL-6, IL-8, IL-10, INF-ɣ, and IL-1β. However, the authors performed a quantitative data synthesis without characterizing different exercise modalities. Moreover, the authors did not exclude patients with advanced or metastatic cancers, with significant implications in terms of inflammatory states, exercise load tolerability, and physical exercise response (Abbasi et al., 2022).

Interestingly, our findings underlined significant effects in the studies assessing CET (Dolan et al., 2010; Kim et al., 2017; Dieli-Conwright et al., 2018a; Dieli-Conwright et al., 2018b) or HIIT protocols (Alizadeh et al., 2019; Hiensch et al., 2021). To the best of our knowledge, this is the first systematic review underlining biomarker modifications induced by different exercise modalities in BC survivors, in contrast with the current trend that considered physical exercises as unicum. On the other hand, our data showed several differences between different physical exercise programs, suggesting the need to better characterize exercise modalities in order to set up the most effective exercise programs for cancer patients. Moreover, it should be noted that chronic inflammation has been previously related to the oncogenesis process through both genetic and epigenetic mechanisms (Trinchieri, 2012; Zitvogel et al., 2017; Barabutis et al., 2018). Several inflammatory mediators have been proposed to have a crucial role in cellular growth and survival, or might directly or indirectly activate oncogenic transcription factors, (including NF-κB and STAT3), or oncogenes (including Ras and Myc) (Karin, 2006; Ancrile et al., 2007; Yu et al., 2007; Mantovani et al., 2008; Grivennikov et al., 2009).

In this scenario, our data underlined significant effects of physical exercise on specific inflammatory biomarkers, highlighting that specific exercise modalities might have an impact on both inflammation and immune response. These data are intriguing in the context of the clinical management of BC patients since the widely noted role of inflammation in the complex process underpinning oncogenesis and cancer growth promotion (Mahadevan and Zanetti, 2011; Koelwyn et al., 2015). In addition, strong evidence supported the role of physical exercise in increasing the overall survival of BC patients, probably due to its effects on oxidative stress and inflammation (Saarto et al., 2012; Winters-Stone et al., 2013; Wennerberg et al., 2020; Delrieu et al., 2021; Vehmanen et al., 2021; Longobucco et al., 2022). Therefore, these biomarker modifications might be the basis for a more precise exercise prescription aiming at reducing systemic inflammation in BC survivors in order to reduce tumor growth or risk of recurrence.

In recent years, an increasing amount of literature has recently underlined that metabolism dysregulation and bioenergetic cell capacity might have a role in all steps of the oncogenesis process, including malignant transformation, tumor progression, and response to cancer treatment (Goodwin et al., 2002; Pasanisi et al., 2006; Gunter et al., 2009; Wallace, 2012; Nelson et al., 2016; Vyas et al., 2016; Kang et al., 2017; Porporato et al., 2018). Moreover, several studies highlighted that high fasting insulin levels have been associated with distant BC recurrence and mortality (Goodwin et al., 2002; Pasanisi et al., 2006; Gunter et al., 2009). Interestingly, our data underlined that physical exercise intervention might significantly improve metabolism biomarkers, leading to benefits in glycemic and insulin profiles, lipid profile, and other anabolic biomarkers.

Our results are in accordance with the systematic review by Kang et al. (2017) published in 2017, reporting that physical exercise might reduce fasting insulin levels in breast cancer survivors. However, the authors failed to demonstrate significant effects in terms of other relevant metabolism biomarkers (Kang et al., 2017). However, in the past few years, recent studies included in our review reported potential effects of physical exercise on several biomarkers involved in the oncogenesis process (Dieli-Conwright et al., 2018a; Dieli-Conwright et al., 2018b; de Paulo et al., 2018; Hartman et al., 2019; Lee et al., 2019; Chang et al., 2020). In this scenario, interventions altering these complex pathways involved in bioenergetic capacity, anabolic processes, and cell homeostasis might be promising targets for the future development of novel anticancer therapies (Porporato et al., 2018). Thus, future research might clarify the biological effects of physical exercise in altering specific metabolic functions, focusing on the reciprocal and multilevel interactions with other environmental stressors that might crucially affect exercise’s biological effects.

Unfortunately, the data reported in our systematic review highlighted little evidence in the exercise-induced bioregulation of cardiac biomarkers. Nevertheless, several studies reported that cTnT might increase as a physiological acute response to exercise (Shin and Cheong, 2019; Cirer-Sastre et al., 2021). Indeed, it has been proposed that the acute release of cTnT might be related to a transient and reversible change in membrane permeability of the myocytes rather than irreversible damage due to myocytes’ necrosis (Middleton et al., 2008). On the other hand, the studies considered did not report differences between groups after the exercise intervention suggesting exercise during cardiotoxic chemotherapy is not associated with increased risks of myocardial damage. Surprisingly, Ansund et al. (2021) reported potential long terms benefits in terms of Nt-pro-BNP in breast cancer patients receiving RET combined with HIIT compared to usual care, suggesting a protective effect. However, there is a large gap of knowledge in the current literature, and further studies with longer follow ups are warranted to clarify the role of exercise training and the optimal modality for mitigating the cardiotoxic effects of chemotherapy.

Taken together, our data underlined several biomarkers involved in bioenergetic metabolism, immune system modulation, and inflammation that might be affected by physical exercise. In the era of precision medicine, an innovative rehabilitation approach based on biomarker modification might be considered in order to maximize outcomes and to focus resources on specific rehabilitative exercise interventions.

We are aware that this systematic review is not free from study limitations. In particular, considering the heterogeneity of the interventions and outcomes assessed, a meta-analysis has not been performed, in accordance with the Cochrane Handbook for Systematic Review of Intervention (Ver, 6.1, 2020) (Higgins JPT). Moreover, the quality assessment might be affected by the lack of blinding of participants or personnel due to the specific characteristics of exercise rehabilitation that cannot be blinded. However, this intrinsic limitation has been shown by the Jadad scale.

However, to the best of our knowledge, this is the first systematic review currently available that highlights the effects of different exercise modalities in biomarkers modifications, emphasizing the biological differences induced by specific exercise programs. These results might be a catalyst for future research focusing on the precise prescription of individualized rehabilitation programs promoting specific biological modifications in BC women.

## 5 Conclusion

To date, rehabilitation has been suggested as an effective non-pharmacological intervention to improve outcomes in BC patients; however, in the era of precision medicine, the optimal biomarkers to assess biological effects of physical exercise are far from being fully understood.

Rehabilitation might have a crucial role not only in the complex management of physical and psychological impairment related to BC and its treatment, but also might counteract the pathological pathways involved in malignant transformation, tumor progression, and response to cancer treatments.

Taken together, the findings of this systematic review showed the importance of several inflammatory and metabolic biomarkers to assess rehabilitation biological effects, paving the way to the future concept of a precise prescription of individualized rehabilitation plan that should be tailored to patient’s characteristics.

Future research should focus on the reciprocal and multilevel interactions between biomarkers, rehabilitation programs, and environmental stressors to deeply understand the complex mechanisms underpinning physical exercise macroscopical effects in BC survivors.
