# Supplementary material for: Integrating molecular biomarkers in breast cancer rehabilitation. What is the current evidence? A systematic review of randomized controlled trials
Source: Front Mol Biosci. 2022 Sep 8;9:930361. doi: 10.3389/fmolb.2022.930361 (PMC9493088; doi:10.3389/fmolb.2022.930361)
Supplement: Supplementary file 1 [file Table1.DOCX]

Supplementary Material

| **Supplementary Table 1.** Search strategy. |
| --- |
| ***PubMed:***  ("breast neoplasms"[MeSH Terms] OR ("breast"[All Fields] AND "neoplasms"[All Fields]) OR "breast neoplasms"[All Fields] OR ("breast"[All Fields] AND "cancer"[All Fields]) OR "breast cancer"[All Fields] OR "breast tumor"[MeSH Terms] OR ("breast"[All Fields] AND "tumor"[All Fields]) OR "breast tumor"[All Fields]) AND ("rehabilitant"[All Fields] OR "rehabilitants"[All Fields] OR "rehabilitate"[All Fields] OR "rehabilitated"[All Fields] OR "rehabilitates"[All Fields] OR "rehabilitating"[All Fields] OR "rehabilitation"[MeSH Terms] OR "rehabilitation"[All Fields] OR "rehabilitations"[All Fields] OR "rehabilitative"[All Fields] OR "rehabilitation"[MeSH Subheading] OR "rehabilitation s"[All Fields] OR "rehabilitational"[All Fields] OR "rehabilitator"[All Fields] OR "rehabilitators"[All Fields] OR "exercise"[MeSH Terms] OR "exercise"[All Fields] OR ("physical"[All Fields] AND "exercise"[All Fields]) OR "physical exercise"[All Fields] OR "training"[All Fields] OR "train"[All Fields] OR "train s"[All Fields] OR "trained"[All Fields] OR "training s"[All Fields] OR "trainings"[All Fields] OR "trains"[All Fields])) AND ("biomarker s"[All Fields] OR "biomarkers"[MeSH Terms] OR "biomarkers"[All Fields] OR "biomarker"[All Fields]) |
| ***Scopus:***  TITLE-ABS-KEY ( ( ( biomarker ) AND ( ( rehabilitation ) OR ( physical AND exercise ) OR ( physical AND activity ) ) AND ( ( breast AND cancer ) OR ( breast AND neoplasms ) OR ( breast AND tumor ) ) ) ) |
| ***Web of Science:***  ALL=((biomarker) AND (rehabilitation OR physical exercise OR physical activity) AND (breast cancer OR breast neoplasm OR breast tumor)) |
| ***Cochrane:***  ID Search  #1 MeSH descriptor: [Biomarker] explode all trees  #2 MeSH descriptor: [Breast cancer] explode all trees  #3 MeSH descriptor: [Breast tumor] explode all trees  #4 MeSH descriptor: [Breast neoplasm] explode all trees  #5 MeSH descriptor: [Rehabilitation] explode all trees  #6 MeSH descriptor: [Physical exercise] explode all trees  #7 MeSH descriptor: [Physical activity] explode all trees  #8 #1 AND (#2 OR #3 OR #4) AND (#5 OR #6 OR #7) |
| ***PEDro:***  Biomarker Breast Cancer Physical exercise |

| Supplementary Table 2. Characteristics of excluded studies assessed in full-text. | |
| --- | --- |
| *Study* | ***Reason for exclusion*** |
| Knobf et al. 2016 | No homogeneous sample of BC |
| Winkels et al. 2017 | Protocol study |
| Tjoe et al. 2020 | Not RCT |
| Bower et al. 2014 | No rehabilitative treatment |
| Villarini et al. 2011 | Protocol study |
| McClain et al. 2021 | No breast cancer patients |
| Reich et al. 2017 | No rehabilitative treatment |
| Bartlett et al. 2021 | Not RCT |
| Eremin et al. 2009 | No rehabilitative treatment |
| Evans et al. 2016 | Not RCT |
| Natalucci et al. 2021 | Not RCT |
| Rogers et al. 2009 | No relevant biomarkers |
| Adams et al. 2018 | Included other intervention |
| Puklin et al. 2020 | Included other intervention |
| Sanft et al. 2018 | Included other intervention |
| Scott et al. 2013 | Included other intervention |
| Swinsher et al 2015 | Included other intervention |
| Demark-Wahnefried et al. 2019 | Included other intervention |
| Toohey et al. 2020 | No relevant biomarkers |
| D’Alonzo et al. 2021 | Included other intervention |
| Sturgeon et al. 2018 | Included other intervention |
| Febvey-Combes et al. 2021 | Included other intervention |
| Baker et al. 2018 | No relevant biomarkers |
| Waltman et al. 2010 | No relevant biomarkers |
| Winters-Stone et al. 2011 | No relevant biomarkers |
| Parma et al. 2015 | Not specified cancer stage |
| Payne et al. 2008 | Not specified cancer stage |
| Bruno et al. 2016 | Included other intervention |
| Dieli‑Conwright et al. 2021 | Not RCT |
| Boyne et al. 2018 | No breast cancer patients |
| Gonzalo-Encabo et al. 2021 | No breast cancer patients |
| Artene et al. 2017 | No relevant biomarkers |
| Casla et al. 2015 | No relevant biomarkers |
| Courneya et al. 2007 | No relevant biomarkers |
| Courneya et al. 2014 | No relevant biomarkers |
| Bao et al. 2015 | Not RCT |
| Djuric et al. 2012 | Not RCT |
| Fabian et al. 2021 | Not RCT |
| Howden et al. 2019 | Not RCT |
| Su et al. 2016 | Not RCT |
| Zimmer et al. 2018 | Not RCT |
| Toriola et al. 2015 | No rehabilitative treatment |
| Brown et al. 2020 | No breast cancer patients |
| Karimi et al. 2013 | No homogeneous sample of BC |
| Van Gemert et al. 2016 | Included other intervention |
| Winters Stone et al. 2017 | Not RCT |
| Hutnick et al. 2005 | Not RCT |
| Ligibel et al. 2019 | Pre-Operative intervention |
