# Supplementary material for: Integrating molecular biomarkers in breast cancer rehabilitation. What is the current evidence? A systematic review of randomized controlled trials
Source: Front Mol Biosci. 2022 Sep 8;9:930361. doi: 10.3389/fmolb.2022.930361 (PMC9493088; doi:10.3389/fmolb.2022.930361)
Supplement: Supplementary file 2 [file Table2.DOCX]

Supplementary Material

| Supplementary Table 2. Characteristics of excluded studies assessed in full-text. | |
| --- | --- |
| *Study* | ***Reason for exclusion*** |
| Knobf et al. 2016 | No homogeneous sample of BC |
| Winkels et al. 2017 | Protocol study |
| Tjoe et al. 2020 | Not RCT |
| Bower et al. 2014 | No rehabilitative treatment |
| Villarini et al. 2011 | Protocol study |
| McClain et al. 2021 | No breast cancer patients |
| Reich et al. 2017 | No rehabilitative treatment |
| Bartlett et al. 2021 | Not RCT |
| Eremin et al. 2009 | No rehabilitative treatment |
| Evans et al. 2016 | Not RCT |
| Natalucci et al. 2021 | Not RCT |
| Rogers et al. 2009 | No relevant biomarkers |
| Adams et al. 2018 | Included other intervention |
| Puklin et al. 2020 | Included other intervention |
| Sanft et al. 2018 | Included other intervention |
| Scott et al. 2013 | Included other intervention |
| Swinsher et al 2015 | Included other intervention |
| Demark-Wahnefried et al. 2019 | Included other intervention |
| Toohey et al. 2020 | No relevant biomarkers |
| D’Alonzo et al. 2021 | Included other intervention |
| Sturgeon et al. 2018 | Included other intervention |
| Febvey-Combes et al. 2021 | Included other intervention |
| Baker et al. 2018 | No relevant biomarkers |
| Waltman et al. 2010 | No relevant biomarkers |
| Winters-Stone et al. 2011 | No relevant biomarkers |
| Parma et al. 2015 | Not specified cancer stage |
| Payne et al. 2008 | Not specified cancer stage |
| Bruno et al. 2016 | Included other intervention |
| Dieli‑Conwright et al. 2021 | Not RCT |
| Boyne et al. 2018 | No breast cancer patients |
| Gonzalo-Encabo et al. 2021 | No breast cancer patients |
| Artene et al. 2017 | No relevant biomarkers |
| Casla et al. 2015 | No relevant biomarkers |
| Courneya et al. 2007 | No relevant biomarkers |
| Courneya et al. 2014 | No relevant biomarkers |
| Bao et al. 2015 | Not RCT |
| Djuric et al. 2012 | Not RCT |
| Fabian et al. 2021 | Not RCT |
| Howden et al. 2019 | Not RCT |
| Su et al. 2016 | Not RCT |
| Zimmer et al. 2018 | Not RCT |
| Toriola et al. 2015 | No rehabilitative treatment |
| Brown et al. 2020 | No breast cancer patients |
| Karimi et al. 2013 | No homogeneous sample of BC |
| Van Gemert et al. 2016 | Included other intervention |
| Winters Stone et al. 2017 | Not RCT |
| Hutnick et al. 2005 | Not RCT |
| Ligibel et al. 2019 | Pre-Operative intervention |
