# Supplementary material for: Integrating molecular biomarkers in breast cancer rehabilitation. What is the current evidence? A systematic review of randomized controlled trials
Source: Front Mol Biosci. 2022 Sep 8;9:930361. doi: 10.3389/fmolb.2022.930361 (PMC9493088; doi:10.3389/fmolb.2022.930361)
Supplement: Supplementary file 3 [file Table3.docx]

Supplementary Material

| **Supplementary Table 3.** Main characteristics of the studies included. | | | | | | | | |
| --- | --- | --- | --- | --- | --- | --- | --- | --- |
| **Authors**  **Journal**  **Year** | **Participants** | | | **Intervention** | **Comparator** | **Results** | | |
|  | ***Sample Characteristics*** | ***Cancer Characteristics*** | ***Cancer Treatments*** |  |  | ***Intragroup Analysis - IG*** | ***Intragroup Analysis - CG*** | ***Intergroups Analysis*** |
| Alizadeh et al.  *J Cancer Res Clin Oncol.*  2019 | N = 50  IG: 24  CG: 26  *Mean Age (years)*  IG: 49.2±9.7  CG: 48.42±7.54  *BMI (kg/m2)*  IG: 27.85±4.01  CG: 27.98±3.90 | Stages I- III, hormone-responsive breast cancer  *T1*  IG: 9, CG: 6  *T2*  IG: 10, CG: 9  *T3*  IG: 6, CG: 8  *T4*  IG: 10, CG: 8  *N0*  IG: 13, CG: 14  *N1*  IG: 2, CG: 1 | *Chemotherapy*  Completed  IG: 17/24  CG: 16/26  *Radiotherapy*  Completed  IG: 11/24  CG: 13/26  *Hormonal Therapy*  100% | *Type of activity:* HIIT  *Exercise modality:* 4×4 min of uphill walking (exercise) and 4×3 min of uphill walking (active recovery) on motorized treadmill  *Protocol duration:* 12 weeks  *Frequency:* 3 session/wk  *Volume (session):* 38 min (5 min of warm-up, 5 min of cool down, 16 min of high-intensity interval and 12 min of active recovery between intervals)  *Intensity:* 90–95% HRmax (exercise) and 50–70% HRmax (active recovery)  *Supervised*  *After chemotherapy and/or radiotherapy treatments* | Usual care | NR | NR | *Inflammation Biomarkers*  TNF-α: mean values = NR; p = 0.001  IL-6: mean values = NR; p = 0.007  IL-10: mean values = NR; p = 0.001  IL-1β: mean values = NR; p = 0.093  TNF-α/IL-10 ratio: mean values = NR; p = 0.050  IL-6/IL-10: mean values = NR; p = 0.042  IL-4: mean values = NR; p = 0.050  IFN-γ: mean values = NR; p = 0.660  HSP70: mean values = NR; p = 0.050 |
| Ansund et al.  *Cardiooncology*  2021 | N = 88  RET-HIIT: 29  AT–HIIT: 32  CG: 27  *Mean Age (years)*  RET-HIIT: 53.5±10.2  AT–HIIT: 53.7±7.9  CG: 55.9±7.5  *BMI (kg/m2)*  RET-HIIT: 24.0±2.9  AT–HIIT: 24.2±3.3  CG: 25.4±4.4 | Stages I–IIIa breast cancer | *Chemotherapy*  100%  *Radiotherapy*  RET-HIIT: 86.2%  AT–HIIT: 87.5%  CG: 85.2% | RET-HIIT:  *Type of activity:* resistance and HIIT  *Exercise modality:* RET targeting the major muscle groups, followed by aerobic HIIT on a cycle ergometer  *Protocol duration:* 16 weeks  *Frequency:* 2 session/wk  *Volume (session):* 8–12 Rep + 3×3 min bouts of aerobic HIIT  *Intensity:* 75–80% of 1RM (RET) and NR for HIIT  *Supervised*  *During cancer treatments*  AT–HIIT:  *Type of activity:* moderate-intensity aerobic and HIIT  *Exercise modality:* moderate intensity continuous AET followed by aerobic HIIT on a cycle ergometer  *Protocol duration:* 16 weeks  *Frequency:* 2 session/wk  *Volume (session):* 20 minutes (AET) + 3×3 min bouts of aerobic HIIT  *Intensity:* NR for AET and NR for HIIT  *Supervised*  *During cancer treatments* | Exercise recommendations according to 2010 ACSM exercise guidelines | *Cardiac Biomarkers*  At 16-week, RET-HIIT and AT–HIIT  cTnT mean values = NR; p < 0.05  Nt-pro-BNP mean values = NR; p > 0.05 | *Cardiac Biomarkers*  At 16-week  cTnT mean values = NR; p < 0.05  Nt-pro-BNP mean values = NR; p > 0.05 | *Cardiac Biomarkers*  At 16-week, between all groups  cTnT mean values = NR; p > 0.05  Nt-pro-BNP mean values = NR; p > 0.05  At 1 year, CG vs RET-HIIT and CG vs AET-HIIT  Nt-pro-BNP mean values = NR; p = 0.036 |
| Chang et al.  *Sci Rep.*  2020 | N = 34  IG: 17  CG: 17  *Mean Age (years)*  IG: 51.4±7.5  CG: 50.0±6.1  *BMI (kg/m2)*  IG: 22.7±2.6  CG: 24.6±4.4 | Stage I‒III breast cancer | NR | *Type of activity:* combined training  *Exercise modality:* warm-up (stretching and pendulum exercises) + CET [step aerobics using height-adjustable platforms combined with progressive strength training using various body weights and elastic bands (shoulder presses, black burn exercise, wall push-ups, biceps curls, planks, leg bridges, squats, and calf raises)] + cool-down (easy walking and stretching)  *Protocol duration:* 12 weeks  *Frequency:* 3 session/wk  *Volume (session):* 10 minutes of warm-up, 40 minutes of CET (RET: 3 sets of 12‒16 reps, for each exercise) + 10 minutes of cool-down.  *Intensity:* AET: 11-13 RPE at the start to 13-15 RPE at the end; RET: 6–8 on the OMNI-RES (60-80% 1RM)  *Supervised*  *After chemotherapy and/or radiotherapy treatments* | No intervention | *Metabolism Biomarkers*  Triglyceride (mg/dL)  110.0 ± 49.9 ± 28.1 vs 102.1 ± 50.5; p = 0.099  Total cholesterol (mg/dl)  192.6 ± 28.1 vs 178.8 ± 27.9; p = 0.023  LDL (mg/dl)  114.3 ± 31.0 vs 99.5 ± 26.7; p = 0.018  Glucose (mg/dL)  88.6 ± 14.8 vs 92.2 ± 6.8; p = 0.358  Fasting insulin (μIU)  10.9 ± 6.8 vs 8.1 ± 3.4; p = 0.035  HOMA1-IR (μU/ml) × (mg/dl)  2.47 ± 1.46 vs 1.83 ± 0.80; p = 0.025  HOMA2-IR (μU/ml) × (mg/dl)  1.40 ± 0.84 vs 1.05 ± 0.44; p = 0.035  *Tumor-metabolism Biomarker*  β-catenin (pg/mL)  53.4 ± 15.4 vs 44.5 ± 10.2; p = 0.046  WISP-1 (pg/mL)  115.9 ± 45.9 vs 97.4 ± 33.7; p = 0.031 | *Metabolism Biomarkers*  Triglyceride (mg/dL)  116.6 ± 65.0 vs 131.9 ± 91.7; p = 0.583  Total cholesterol (mg/dl)  181.4 ± 33.2 vs 178.2 ± 36.5; p = 0.547  LDL (mg/dl)  104.8 ± 30.0 vs 96.7 ± 30.2; p = 0.116  Glucose (mg/dL)  95.3 ± 21.3 vs 98.1 ± 23.9; p = 0.184  Fasting insulin (μIU)  13.1 ± 10.2 vs 12.5 ± 11.0; p = 0.492  HOMA1-IR (μU/ml) × (mg/dl)  3.11 ± 2.69 vs 3.06 ± 2.92; p = 0.653  HOMA2-IR (μU/ml) × (mg/dl)  1.68 ± 1.31 vs 1.62 ± 1.41; p = 0.463  *Tumor-metabolism Biomarker*  β-catenin (pg/mL)  49.9 ± 15.0 vs. 49.7 ± 13.2; p = 0.938  WISP-1 (pg/mL)  110.1 ± 36.8 vs. 105.4 ± 41.1; p = 0.407 | NR |
| De Paulo et al.  *Exp Gerontol.*  2018 | N = 36  IG: 18  CG: 18  *Mean Age (years)*  IG: 63.2±7.1  CG: 66.6±9.6  *BMI (kg/m2)*  IG: 28.9±5.2  CG: 31.5±6.3 | Stage I–IIIA breast cancer  *I*  IG: 50%  CG: 58.8%  *II*  IG: 33.3%  CG: 23.6%  *III*  IG: 17.6%  CG: 17.6% | *Lumpectomy*  IG: 47.4%  CG: 58.8%  *Mastectomy*  IG: 52.6%  CG: 41.2%  *Hormonal Therapy*  100% | *Type of activity:* CET  *Exercise modality:* warm-up, RET on weight machines (seated cable row, bench press, leg extension, leg press, and leg curl, as well as bridge, abdominal, and plank exercises), AET on treadmill, cool-down  *Protocol duration:* 36 weeks  *Frequency:* 3 session/wk  *Volume (session):* 100 min of CET (5 min warm-up, approximately 55 min of RET, 30 min of AET, 10 min cool-down)  *Intensity:* NR  *Supervised*  *After chemotherapy and/or radiotherapy treatments* | *Type of activity:* low-intensity stretching and relaxation exercises  2 session/wk  45 min per sessions  36 weeks | *Metabolism Biomarkers*  Cholesterol (mg/dl)  219 ± 41 vs 213 ± 35; p = NS  HDL (mg/dl)  55 ± 10 vs 51 ± 8; p = NS  LDL (mg/dl)  135 ± 37 vs 126 ± 26; p = NS  Triglycerides (mg/dl)  148 ± 68 vs 147 ± 55; p = NS  Glucose (mg/dl)  107 ± 24 to 103 ± 19; p = NS  *Inflammation Biomarkers*  CRP (mg/dl)  5 ± 4 to 4 ± 3; p = NS | *Metabolism Biomarkers*  Cholesterol (mg/dl)  207 ± 41 vs 211 ± 46; p = NS  HDL (mg/dl)  53 ± 10 vs 52 ± 10; p = NS  LDL (mg/dl)  131 ± 36 vs 137 ± 42; p = NS  Triglycerides (mg/dl)  136 ± 41 vs 145 ± 43; p = NS  Glucose (mg/dl)  110 ± 22 to 108 ± 19; p = NS  *Inflammation Biomarkers*  CRP (mg/dl)  7 ± 7 to 6 ± 6; p = NS | *Metabolism Biomarkers*  Cholesterol (mg/dl)  IG: 213 ± 35 vs CG: 211 ± 46; p= 0.51  HDL (mg/dl)  IG: 51 ± 8 vs CG: 52 ± 10; p= 0.20  LDL (mg/dl)  IG: 126 ± 26 vs CG: 137 ± 42; p= 0.06  Triglycerides (mg/dl)  IG: 147 ± 55 vs CG: 145 ± 43; p= 0.83  Glucose (mg/dl)  IG: 103 ± 19 vs CG: 108 ± 19; p= 0.41  *Inflammation Biomarkers*  CRP (mg/dl)  IG: 4 ± 3 vs CG: 6 ± 6; p= 0.05 |
| Dieli-Conwright et al.  *Breast Cancer Res Treat.*  2018 | N = 20  IG: 10  CG: 10  *Mean Age (years)*  IG: 53.0±10.0  CG: 55.0±4.5  *BMI (kg/m2)*  IG: 33.5±5.7  CG: 33.3±8.7 | Stage I-III breast cancer  *I*  IG: 3 (30%)  CG: 3 (30%)  *II*  IG: 4 (40%)  CG: 4 (40%)  *III*  IG: 3 (30%)  CG: 3 (30%) | *Lumpectomy*  IG: 3 (30%)  CG: 2 (20%)  *Mastectomy*  IG: 7 (70%)  CG: 8 (80%)  *Chemotherapy*  IG: 8 (80%)  CG: 7 (70%)  *Radiotherapy*  IG: 5 (50%)  CG: 6 (60%) | *Type of activity:* CET  *Exercise modality:* warm-up (AET), RET (leg press, lunges, leg extension, leg flexion, chest press, seated row, triceps extension, and biceps curl) performed in a circuit training, AET self-selecting their preferred mode of exercise (treadmill walking/running,  rowing machine or stationary bicycle), cool-down (AET)  *Protocol duration:* 16 weeks  *Frequency:* 2 session/wk of CET, and 1 session/wk of AET  *Volume (session):* CET: 80 min, and AET: 50 min. warm-up = 5 min; RET = 3 sets of  10-15 reps; AET = 30-50 min; cool-down = 5 min  *Intensity:* warm-up = 40-50% VO2max; RET = 80% 1-RM (lower body) 60% 1-RM (upper body); AET = 65-80% HRmax; cool-down = 40-50% VO2max  *Supervised*  *After chemotherapy and/or radiotherapy treatments* | Usual care | *Metabolism Biomarkers*  Glucose (mg/dl)  Pre: 109.6 ± 17.6 Mean change: −12.0 ± 16.0; p = 0.016  Fasting insulin (uIU/mL)  Pre: 35.0 ± 22.2 Mean change: −13.5 ± 13.8; p= 0.002  HOMA-IR  Pre: 10.04 ± 8.80 Mean change: −4.91 ± 6.93; p = 0.002  Total cholesterol (mg/dl)  Pre: 190.5 ± 50.40 Mean change: −39.5 ± 32.7; p = 0.004  LDL (mg/dl)  Pre: 102.9 ± 34.4 Mean change: −25.0 ± 23.2; p = 0.008  HDL (mg/dl)  Pre: 43.2 ± 6.6 Mean change: 20.2 ± 8.2; p = 0.002  Triglycerides (mg/dl)  Pre: 242.6 ± 26.8 Mean change: −97.7 ± 27.2; p = 0.002  HbA1c (%)  Pre: 5.48 ± 0.44 Mean change: −1.74 ± 0.72; p = 0.002  *Inflammation Biomarkers*  CPR (mg/dl)  Pre: 3.20 ± 0.64 Mean change: −0.92 ± 0.72; p = 0.008  Leptin (ng/dl)  Pre: 12.2 ± 2.0 Mean change: −3.0 ± 1.9; p = 0.008  Adiponectin (ng/ml)  Pre: 13.2 ± 1.7 Mean change: 8.7 ± 2.1; p = 0.002  IL-6 (ng/dL)  Pre: 3.15 ± 0.49 Mean change: 3.15 ± 0.49; p = 0.002  IL-8 (ng/dl)  Pre: 5.53 ± 1.00 Mean change: −1.28 ± 0.80; p = 0.002  M1 (%) from adipose tissue biopsy  Pre: 24.0 ± 6.2 Mean change: −17.4 ± 7.1; p = 0.002  M2 (%) from adipose tissue biopsy  Pre: 4.0 ± 1.3 Mean change: 9.0 ± 1.6; p = 0.002  Adiponectin (ng/mL) from adipose tissue biopsy  Pre: 91.68 ± 21.05 Mean change: 46.61 ± 25.19; p = 0.03  IL-12 p40 (pg/mL) from adipose tissue biopsy  Pre: 8.83 ± 13.13 Mean change: 1.93 ± 20.18; p = 0.75  IL-12 p70 (pg/mL) from adipose tissue biopsy  Pre: 2.53 ± 1.06 Mean change: −1.02 ± 2.12; p = 0.28 | *Metabolism Biomarkers*  Glucose (mg/dl)  Pre: 94.7 ± 14.0 Mean change: 4.1 ± 6.6; p = 0.12  Fasting insulin (uIU/mL)  Pre: 31.9 ± 5.7 Mean change: 7.6 ± 4.2; p = 0.004  HOMA-IR  Pre: 7.50 ± 2.09 Mean change: 2.20 ± 0.99; p = 0.004  Total cholesterol (mg/dl)  Pre: 189.4 ± 18.9 Mean change: 16.4 ± 16.0; p = 0.004  LDL (mg/dl)  Pre: 89.2 ± 18.8 Mean change: 25.6 ± 25.9; p = 0.008  HDL (mg/dl)  Pre: 41.0 ± 4.3 Mean change: 1.1 ± 2.1; p = 0.25  Triglycerides (mg/dl)  Pre: 229.7 ± 24.6 Mean change: 1.0 ± 3.2; p = 1.0  HbA1c (%)  Pre: 5.30 ± 0.48 Mean change: 0.72 ± 0.70; p = 0.016  *Inflammation Biomarkers*  CPR (mg/dl)  Pre: 4.01 ± 0.25 Mean change: 0.40 ± 0.84; p = 0.5  Leptin (ng/dl)  Pre: 12.5 ± 2.0 Mean change: 1.9 ± 1.6; p = 0.016  Adiponectin (ng/ml)  Pre: 13.0 ± 1.8 Mean change: −1.6 ± 2.8; p = 0.13  IL-6 (ng/dL)  Pre: 3.23 ± 0.51 Mean change: 0.42 ± 0.35; p = 0.004  IL-8 (ng/dl)  Pre: 5.46 ± 0.62 Mean change: 0.48 ± 0.56; p = 0.016  M1 (%) from adipose tissue biopsy  Pre: 26.3 ± 7.9 Mean change: 3.0 ± 4.8; p = 0.13  M2 (%) from adipose tissue biopsy  Pre: 4.3 ± 1.1 Mean change: −0.4 ± 1.0; p = 0.31  Adiponectin (ng/mL) from adipose tissue biopsy  Pre: 88.58 ± 37.81 Mean change: −13.83 ± 26.03; p = 0.31  IL-12 p40 (pg/mL) from adipose tissue biopsy  Pre: 11.48 ± 19.45 Mean change: 50.36 ± 44.57; p = 0.13  IL-12 p70 (pg/mL) from adipose tissue biopsy  Pre: 1.70 ± 1.32 Mean change: 1.56 ± 1.62; p = 0.063 | *Metabolism Biomarkers*  Glucose (mg/dl)  IG mean change: −12.0 ± 16.0 vs CG mean change: 4.1 ± 6.6; p = 0.0012  Fasting insulin (uIU/mL)  IG mean change: −13.5 ± 13.8 vs CG mean change: 7.6 ± 4.2; p = 0.0001  HOMA-IR  IG mean change: −4.91 ± 6.93 vs CG mean change: 2.20 ± 0.99; p = 0.0002  Total cholesterol (mg/dl)  IG mean change: −39.5 ± 32.7 vs CG mean change: 16.4 ± 16.0; p = 0.0002  LDL (mg/dl)  IG mean change: −25.0 ± 23.2 vs CG mean change: 25.6 ± 25.9; p = 0.0003  HDL (mg/dl)  IG mean change: 20.2 ± 8.2 vs CG mean change: 1.1 ± 2.1; p = 0.0001  Triglycerides (mg/dl)  IG mean change: −97.7 ± 27.2 vs CG mean change: 1.0 ± 3.2; p = 0.0001  HbA1c (%)  IG mean change: −1.74 ± 0.72 vs CG mean change: 0.72 ± 0.70; p = 0.0002  *Inflammation Biomarkers*  CPR (mg/dl)  IG mean change: −0.92 ± 0.72 vs CG mean change: 0.40 ± 0.84; p = 0.0008  Leptin (ng/dl)  IG mean change: −3.0 ± 1.9 vs CG mean change: 1.9 ± 1.6; p = 0.0004  Adiponectin (ng/ml)  IG mean change: 8.7 ± 2.1 vs CG mean change: −1.6 ± 2.8; p = 0.0001  IL-6 (ng/dL)  IG mean change: −1.02 ± 0.53 vs CG mean change: 0.42 ± 0.35; p = 0.0002  IL-8 (ng/dl)  IG mean change: −1.28 ± 0.80 vs CG mean change: 0.48 ± 0.56; p = 0.0002  M1 (%) from adipose tissue biopsy  IG mean change: −17.4 ± 7.1 vs CG mean change: 3.0 ± 4.8; p = 0.0001  M2 (%) from adipose tissue biopsy  IG mean change: −0.4 ± 1.0 vs CG mean change: −0.4 ± 1.0; p = 0.0002  Adiponectin (ng/mL) from adipose tissue biopsy  IG mean change: 46.61 ± 25.19 vs CG mean change: −13.83 ± 26.03; p = 0.008  IL-12 p40 (pg/mL) from adipose tissue biopsy  IG mean change: 1.93 ± 20.18 vs CG mean change: 50.36 ± 44.57; p = 0.055  IL-12 p70 (pg/mL) from adipose tissue biopsy  IG mean change: −1.02 ± 2.12 vs CG mean change: 1.56 ± 1.62; p = 0.08 |
| Dieli-Conwright et al.  *J Clin Oncol.*  2018 | N = 100  IG: 50  CG: 50  *Mean Age (years)*  N = 53.5±10.4  IG: 52.8±10.6  CG: 53.6±10.1  *BMI (kg/m2)*  N = 33.2±5.5  IG: 33.1±5.7  CG: 33.4±5.2 | Stage I-III primary breast cancer  *I*  IG: 20 (40%)  CG: 21 (42%)  *II*  IG: 19 (38%)  CG: 19 (38%)  *III*  IG: 11 (22%)  CG: 10 (20%) | *Chemotherapy*  IG: 8 (15%)  CG: 5 (11%)  *Radiotherapy*  IG: 4 (8%)  CG: 6 (13%)  *Chemotherapy*  and *Radiotherapy*  IG: 38 (76%)  CG: 38 (70%)  *Hormonal Therapy*  IG: 44 (88%)  CG: 40 (80%) | *Type of activity:* CET  *Exercise modality:* warm-up (AET), RET (leg press, lunges, leg extension, leg flexion, chest press, seated row, triceps extension, and biceps curl) performed in a circuit training, AET self-selecting their preferred mode of exercise (treadmill walking/running, rowing machine, or stationary bicycle), cool-down (AET)  *Protocol duration:* 16 weeks  *Frequency:* 2 session/wk of CET, and 1 session/wk of AET  *Volume (session):* CET: 80 min, and AET: 50 min (warm-up = 5 min; RET = 3 sets of  10-15 reps; AET = 30-50 min; cool-down = 5 min)  *Intensity:* warm-up = 40-50% VO2max; RET = 80% 1-RM (lower body) 60% 1-RM (upper body); AET = 65-80% HRmax; cool-down = 40-50% VO2max  *Supervised*  *After chemotherapy and/or radiotherapy treatments* | Usual care | Baseline vs Post intervention  Baseline vs 3-Month Follow-Up  *Metabolism Biomarkers*  HDL (mg/dl)  43.1 (6.6) vs 64.7 (7.8); p = 0.001  43.1 (6.6) vs 57.4 (7.5); p = 0.001  Triglycerides (mg/dl)  248.1 (26.9) vs 151.8 (19.2); p < .001  248.1 (26.9) vs 159.4 (20.1); p = 0.001  Glucose (mg/dl)  109.6 (17.7) vs 93.0 (12.1); p = .0002  109.6 (17.7) vs 96.1 (13.5); p = 0.001  Fasting insulin (mU/mL)  36.1 (18.2) vs 22.2 (10.1); p = 0.001  36.1 (18.2) vs 23.1 (10.5); p = 0.001  HOMA-IR  11.1 (8.9) vs 6.8 (1.3; p = 0.001  11.1 (8.9) vs 7.0 (1.8); p = 0.001  IGF-1 (ng/mL)  118.4 (31.7) vs 106.3 (26.9); p = .001  118.4 (31.7) vs 106.8 (27.3); p = 0.002  IGFBP-3 (ng/mL)  41.4 (3.9) vs 48.3 (4.4); p = 0.001  41.4 (3.9) vs 47.9 (4.1); p = 0.001  Total cholesterol (mg/dL)  196.5 (53.4) vs 157.5 (37.1); p = 0.001  196.5 (53.4) vs 160.0 (36.5); p = 0.001  SHBG (nmol/L)  54.8 (6.7) vs 63.7 (7.5); p = 0.001  54.8 (6.7) vs 62.7 (7.8); p = 0.001  Estradiol (pg/mL)  9.1 (0.9) vs 7.0 (0.5); p = 0.001  9.1 (0.9) vs 7.0 (0.5); p = 0.001  Free tesvssterone (pg/mL)  2.5 (1.8) vs 3.2 (1.3); p = 0.09  2.5 (1.8) vs 3.0 (1.5); p = 0.08  *Inflammation Biomarkers*  CRP (mg/L)  3.4 (0.6) vs 2.3 (0.3); p = 0.001  3.4 (0.6) vs 2.4 (0.4); p = 0.001  Leptin (ng/mL)  32.5 (2.0) vs 24.5 (2.3); p = 0.001  32.5 (2.0) vs 24.8 (2.0); p = 0.003  Adiponectin (mg/mg)  13.6 (1.7) vs 21.1 (2.7); p = 0.001  13.6 (1.7) vs 20.0 (2.8); p = 0.001  IL-6 (pg/mL)  3.3 (0.5) vs 1.9 (0.3); p = 0.001  3.3 (0.5) vs 2.0 (0.2); p = 0.007  IL-8 (pg/mL)  5.5 (1.0) vs 3.7 (0.8); p = 0.001  5.5 (1.0) vs 3.7 (0.8); p = 0.001  TNF-a (pg/mg)  2.4 (0.8) vs 1.2 (0.6); p = 0.001  2.4 (0.8) vs 1.2 (0.5); p = 0.001 | Baseline vs Post intervention  *Metabolism Biomarkers*  HDL (mg/dl)  41.0 (4.3) vs 39.9 (4.0); p = 0.45  Triglycerides (mg/dl)  231.8 (24.1) vs 233.7 (25.6); p = 0.87  Glucose (mg/dl)  103.3 (14.0) vs 108.1 (15.1); p = 0.37  Fasting insulin (mU/mL)  34.9 (15.6) vs 42.5 (17.1); p = 0.002  HOMA-IR  12.3 (7.1) vs 15.1 (6.4); p = 0.001  IGF-1 (ng/mL)  119.0 (33.2) vs 127.1 (34.0); p = 0.11  IGFBP-3 (ng/mL)  40.9 (3.3) vs 39.7 (3.5); p = 0.29  Total cholesterol (mg/dL)  194.4 (48.9) vs 210.4 (52.2); p = 0.31  SHBG (nmol/L)  53.7 (5.9) vs 52.6 (5.6); p = 0.66  Estradiol (pg/mL)  9.3 (1.1) vs 10.6 (1.0); p = 0.28  Free testosterone (pg/mL)  2.8 (1.6) vs 2.3 (1.4); p = 0.10  *Inflammation Biomarkers*  CRP (mg/L)  3.7 (0.3) vs 4.2 (0.4); p = 0.54  Leptin (ng/mL)  33.1 (2.9) vs 37.9 (3.4); p = 0.001  Adiponectin (mg/mg)  13.0 (2.0) vs 12.0 (2.3); p = 0.46  IL-6 (pg/mL)  3.2 (0.5) vs 3.3 (0.6); p = 0.47  IL-8 (pg/mL)  5.4 (0.6) vs 6.6 (0.9); p = 0.001  TNF-a (pg/mg)  2.5 (0.9) vs 2.7 (0.9); p = 0.34 | Between group post intervention  *Metabolism Biomarkers*  HDL (mg/dl)  IG: 64.7 (7.8) vs CG: 39.9 (4.0); p = 0.001  Triglycerides (mg/dl)  IG: 151.8 (19.2) vs CG: 233.7 (25.6); p < 0.001  Glucose (mg/dl)  IG: 93.0 (12.1) vs CG: 108.1 (15.1); p = 0.001  Fasting insulin (mU/mL)  IG: 22.2 (10.1) vs CG: 42.5 (17.1); p = 0.002  HOMA-IR  IG: 6.8 (1.3) vs CG: 15.1 (6.4); p = 0.001  IGF-1 (ng/mL)  IG: 106.3 (26.9) vs CG: 127.1 (34.0); p = 0.001  IGFBP-3 (ng/mL)  IG: 48.3 (4.4) vs CG: 39.7 (3.5); p = 0.001  Total cholesterol (mg/dL)  IG: 157.5 (37.1) vs CG: 210.4 (52.2); p = 0.001  SHBG (nmol/L)  IG: 63.7 (7.5) vs CG: 52.6 (5.6); p = 0.001  Estradiol (pg/mL)  IG: 7.0 (0.5) vs CG: 10.6 (1.0); p = 0.001  Free testosterone (pg/mL)  IG: 3.2 (1.3) vs CG: 2.3 (1.4); p = 0.004  *Inflammation Biomarkers*  CRP (mg/L)  IG: 2.3 (0.3) vs CG: 4.2 (0.4); p = 0.001  Leptin (ng/mL)  IG: 24.5 (2.3) vs CG: 37.9 (3.4); p = 0.001  Adiponectin (mg/mg)  IG: 21.1 (2.7) vs CG: 12.0 (2.3); p = 0.001  IL-6 (pg/mL)  IG: 1.9 (0.3) vs CG: 3.3 (0.6); p = 0.001  IL-8 (pg/mL)  IG: 3.7 (0.8) vs CG: 6.6 (0.9); p = 0.001  TNF-a (pg/mg)  IG: 1.2 (0.6) vs CG: 2.7 (0.9); p = 0.001 |
| Dolan et al.  *Cancer Epidemiol Biomarkers Prev.*  2010 | N = 242  RET: 82  AET: 78  CG: 82  *Mean Age (years)*  N = 49.2 (25-78)  RET: 49.5 (25-76)  AET: 49.0 (30-75)  CG: 49.0 (26-78)  *BMI (kg/m2)*  N = 26.6±5.5  RET: 26.1±5.5  AET: 26.7±5.6  CG: 27.1±5.4 | Stage I-IIIA breast cancer  *I*  RET: 22 (26.8%)  AET: 18 (23.1%)  CG: 20 (24.4%)  *IIa*  RET: 36 (43.9%)  AET: 33 (42.3%)  CG: 30 (36.6%)  *IIb*  RET: 9 (11.0%)  AET: 17 (21.8%)  CG: 22 (26.8%)  *IIIa*  RET: 15 (18.3%)  AET: 10 (12.8%)  CG: 10 (12.2%) | *Breast conservation*  RET: 50 (61.0%)  AET: 44 (56.4%)  CG: 49 (59.8%)  *Chemotherapy*  100% | *Type of activity:* RET  *Exercise modality:* 9 different resistance exercises  *Protocol duration:* the duration of their chemotherapy (mean duration: 17 weeks)  *Frequency:* 3 session/wk  *Volume (session):* 2 sets of 8-12 reps  *Intensity:* 60%-70% 1RM  *Supervised*  *During cancer treatments*  *Type of activity:* AET  *Exercise modality:* cycle ergometer, treadmill, or elliptic trainer  *Protocol duration:* the duration of their chemotherapy (mean duration: 17 weeks)  *Frequency:* 3 session/wk  *Volume (session):* 15 min (at the start) – 45 min (at the end)  *Intensity:* 60% (at the start) -80% (at the end) VO2peak  *Supervised*  *During cancer treatments* | Usual care | *Cardiac Biomarkers*  Hb (g/dL)  RET: 13.6 ± 0.7 to 11.0 ± 1.18; p < 0.001  AET: 13.4 ± 1.2 to 11.9 ± 1.3; p < 0.001 | *Cardiac Biomarkers*  Hb (g/dL)  13.3 ± 1.1 to 11.7 ± 1.1; p < 0.001 | *Cardiac Biomarkers*  Hb (g/dL): mean values = NR; p = 0.34 |
| Fairey et al.  *Cancer Epidemiol Biomarkers Prev.*  2003 | N = 52  IG: 24  CG: 28  *Mean Age (years)*  N = 59±6  IG: 59±5  CG: 58±6  *BMI (kg/m2)*  N = 29.2±6.6  IG: 29.4±7.4  CG: 29.1±6.1 | Stage I-IIIB breast cancer  *I*  IG: 10 (42%)  CG: 11 (39%)  *IIa*  IG: 6 (25%)  CG: 11 (39%)  *IIb*  IG: 6 (25%)  CG: 5 (18%)  *IIIa*  IG: 2 (8%)  CG: 1 (4%) | *Lumpectomy*  IG: 9 (37%)  CG: 15 (54%)  *Mastectomy*  IG: 15 (64%)  CG: 13 (46%)  *Chemotherapy*  IG: 10 (42%)  CG: 11 (39%)  *Radiotherapy*  IG: 16 (67%)  CG: 21 (75%)  *Hormonal Therapy*  IG: 11 (46%)  CG: 13 (46%) | *Type of activity:* AET  *Exercise modality:* AET on recumbent or upright cycle ergometers, with an initial warm-up and final cool-down  *Protocol duration:* 15 weeks  *Frequency:* 3 session/wk  *Volume (session):* Warm-up and cool-down = 5 min; AET = 15 min (weeks 1-3) to 35 min (weeks 13–15).  *Intensity:* Warm-up and cool-down = 50% of peak oxygen consumption; AET = 70-75% of peak oxygen consumption  *Supervised*  *After chemotherapy and/or radiotherapy treatments* | Usual care | NR | NR | *Metabolism Biomarkers*  Fasting insulin (pmol/L)  Mean change: +6.3; 95% CI, -6.1 to +18.8; p = 0.941  Glucose (mmol/L)  Mean change: +0.09; 95% CI, -0.3 to +0.5; p = 0.824  Fasting insulin resistance index  Mean change: +0.4; 95% CI, -0.3 to +0.1; p = 0.247  IGF-I (ng/ml)  Mean change: -7.4; 95% CI, -14.6 to -0.2; p = 0.045  IGF-II (ng/ml)  Mean change: -40.7; 95% CI, -89.7 to +8.3; p = 0.101  IGFBP-1 (ng/ml)  Mean change: +1.4; 95% CI, -8.6 to +11.5; p = 0.774  IGFBP-3 (ng/ml)  Mean change: +180.5; 95% CI, 28.4 to 332.5; p = 0.021  IGF-I: IGFBP-3 molar ratio  Mean change: -0.006; 95% CI, -0.01 to -0.001; p = 0.017 |
| Guinan et al.  *Support Care Cancer.*  2013 | N = 26  IG: 16  CG: 10  *Mean Age (years)*  N = 48.12±8.75  IG: 50.05±8.27  CG: 45.05±9.04  *BMI (kg/m2)*  NR | Stage I-III breast cancer  *I*  IG: 3 (18.8%)  CG: 4 (40%)  *II*  IG: 10 (62.6%)  CG: 3 (30%)  *III*  IG: 3 (18.8%)  CG: 3 (30%) | *Mastectomy*  IG: 7 (43.8%)  CG: 6 (60%)  *Wide local excision*  IG: 9 (56.3%)  CG: 4 (40%)  *Axillary dissection*  IG: 9 (56.3%)  CG: 6 (60%)  *Chemotherapy*  100%  *Radiotherapy*  IG: 15 (93.8%)  CG: 9 (90%)  *Hormonal Therapy*  IG: 11 (68.8%)  CG: 9 (90%) | *Type of activity:* AET  *Exercise modality:* Participants  rotated between three aerobic exercise stations during the class (stationary bike, treadmill, rowing ergometer)  *Protocol duration:* 8 weeks  *Frequency:* 2 session/wk  *Volume (session):* 21 min (week 1) to 42 min (week 8)  *Intensity:* 30-35% HRR (at the start) to 65-85% HRR (at the end)  *Supervised and home-based*  *After chemotherapy and/or radiotherapy treatments* | No intervention | NR | NR | *Metabolism Biomarkers*  Total cholesterol (mmol/L)  IG: 5.8 (1.0) to 5.6 (0.9) vs CG: 4.8 (0.5) to 4.3 (0.6); p = 0.69  HDL (mmol/L)  IG: 1.6 (0.6) to 1.5 (0.3) vs CG: 1.5 (0.5) to 1.4 (0.4); p = 0.96  LDL (mmol/L)  IG: 3.5 (0.9) to 3.3 (0.9) vs CG: 2.6 (0.5) to 2.3 (0.9); p = 0.78  Total cholesterol: HDL ratio  IG: 3.9 (0.9) to 3.8 (1.0) vs CG: 3.4 (0.8) to 3.2 (0.8); p = 0.91  Triglycerides (mmol/L)  IG: 1.4 (0.7) to 1.4 (0.6); CG: 1.5 (0.8) to 1.2 (0.7); p = 0.37  Glucose (mg/dL)  IG: 88.3 (7.7) to 90.4 (9.5) vs CG: 88.9 (7.9) to 91.1 (9.9); p = 0.84  Fasting insulin(mU/L)  IG: 10.5 (4.9) to 10.2 (4.5) vs CG: 6.9 (2.8) to 7.4 (2.8); p = 0.51  HOMA-IR  IG: 2.3 (1.1) to 2.3 (1.0) vs CG: 1.5 (0.6) to 1.7 (0.8); p = 0.66  HBA1c (mmol/mol)  IG: 35.9 (2.3) to 35.7 (2.0) vs CG: 35.6 (2.9) to 36.9 (3.9); p = 0.07  *Inflammation Biomarkers*  CRP (mg/L)  IG: -0.65 (95 % CI, -1.66 to 0.35) vs CG: -0.01 (95 % CI, -0.41 to 0.39); p = 0.69 |
| Hartman et al.  *JMIR Cancer.*  2019 | N = 87  IG: 43  CG: 44  *Mean Age (years)*  IG: 58.2±11.37  CG: 56.2±9.30  *BMI (kg/m2)*  IG: 26.7±6.20  CG: 27.3±6.40 | Stage I-III breast  Cancer  *I*  IG: 27 (63%)  CG: 26 (59%)  *II*  IG: 12 (28%)  CG: 15 (34%)  *III*  IG: 4 (9%)  CG: 3 (7%) | *Chemotherapy*  IG: 23 (54%)  CG: 23 (52%)  *Hormonal Therapy*  IG: 31 (72%)  CG: 30 (68%) | *Type of activity:* AET  *Exercise modality:* walking activity  *Protocol duration:* 12 weeks  *Frequency:* 2 session/wk  *Volume (session):* 150 min/wk  *Intensity:* moderate/vigorous  *Supervised*  *After chemotherapy and/or radiotherapy treatments* | No intervention | NR | NR | *Inflammation Biomarkers*  CRP (mg/L)  Mean difference: 0.253; 95% CI −0.04 to 0.57; p = 0.09  *Brain Biomarkers*  BDNF  Mean difference: 0.092; 95% CI −0.25 to 0.43; p = 0.59  *Metabolism Biomarkers*  HOMA2-IR  Mean difference: 0.05; 95% CI −0.12 to 0.22; p = 0.55 |
| Hiensch et al.  *Med Sci Sports Exerc.*  2020 | N = 86  RET-HIIT: 30  AET-HIIT: 27  CG: 29  *Mean Age (years)*  RET-HIIT: 52.2±10.1  AET-HIIT: 53.9±7.4  CG: 52.9±10.1  *BMI (kg/m2)*  RET-HIIT: 24.2±3.6  AET-HIIT: 24.2±3.4  CG: 24.7±4.4 | Stage I-IIIA breast cancer | *Chemotherapy*  100% | *Type of activity:* RET-HIIT  *Exercise modality:* eight resistance exercises followed by high-intensity intermittent aerobic exercise interspersed with low-intensity active recovery  *Protocol duration:* 16 weeks  *Frequency:* 2 session/wk  *Volume (session):* 2–3 sets of 8–12 reps (RET); 3x3-min bouts (high-intensity intermittent aerobic exercise); 1 min active recovery  *Intensity:* 70-80% 1RM (RET); 16–18 RPE (high-intensity intermittent aerobic exercise); low-intensity active recovery  *Supervised and home-based*  *During cancer treatments*  *Type of activity:* AET-HIIT  *Exercise modality:* moderate-intensity aerobic exercise, followed by high-intensity intermittent aerobic exercise interspersed with low-intensity active recovery  *Protocol duration:* 16 weeks  *Frequency:* 2 session/wk  *Volume (session):* 20 min (moderate-intensity aerobic exercise); 3x3-min bouts (high-intensity intermittent aerobic exercise); 1 min active recovery  *Intensity:* 13–15 RPE (moderate-intensity aerobic exercise); 16–18 RPE (high-intensity intermittent aerobic exercise); low-intensity active recovery  *Supervised*  *During cancer treatments* | Usual care | *Inflammation Biomarkers*  CD40-L  RET-HIIT: 5.86 ± 1.87 to 4.90 ± 1.55; Mean (95% CI) -1.06 (-1.79 to -0.32); p < 0.05  AET-HIIT: 6.07 ± 1.60 to 5.52 ± 1.18; Mean (95% CI) -0.48 (-1.30 to 0.35); p = NS  EGF  RET-HIIT: 7.60 ± 1.54 to 6.65 ± 1.35; Mean (95% CI) -1.06 (-1.76 to -0.36); p < 0.05  AET-HIIT: 7.76 ± 1.50 to 7.14 ± 1.34; Mean (95% CI) -0.52 (-1.38 to 0.34); p = NS  IL-6  RET-HIIT: 2.51 ± 0.83 to 2.54 ± 0.74; Mean (95% CI) -0.01 (-0.39 to 0.37); p = NS  AET-HIIT: 2.85 ± 0.96 to 3.03 ± 0.75; Mean (95% CI) 0.21 (-0.09 to 0.51); p = NS  TRAIL  RET-HIIT: 6.98 ± 0.29 to 7.02 ± 0.39; Mean (95% CI) -0.004 (-0.19 to 0.18); p = NS  AET-HIIT: 7.18 ± 0.55 to 7.33 ± 0.49; Mean (95% CI) 0.14 (-0.03 to 0.30); p = NS  CD8a  RET-HIIT: 8.19 ± 0.86 to 8.00 ± 0.72; Mean (95% CI) -0.24 (-0.44 to -0.03); p < 0.05  AET-HIIT: 8.07 ± 0.70 to 7.96 ± 0.74; Mean (95% CI) -0.12 (-0.29 to 0.06); p = NS  DCN  RET-HIIT: 3.55 ± 0.33 to 3.56 ± 0.39; Mean (95% CI) -0.02 (-0.12 to 0.09); p = NS  AET-HIIT: 3.64 ± 0.50 to 3.75 ± 0.52; Mean (95% CI) 0.08 (-0.03 to 0.20); p = NS  CCL17  RET-HIIT: 7.23 ± 1.28 to 6.85 ± 1.23; Mean (95% CI) -0.45 (-0.94 to 0.03); p = NS  AET-HIIT: 7.28 ± 0.96 to 7.13 ± 1.08; Mean (95% CI) -0.12 (-0.62 to 0.38); p = NS  CASP-8  RET-HIIT: 3.79 ± 1.25 to 3.18 ± 0.91; Mean (95% CI) -0.75 (-1.34 to -0.16); p < 0.05  AET-HIIT: 3.70 ± 1.13 to 3.43 ± 0.74; Mean (95% CI) -0.27 (-0.89 to 0.35); p = NS  ICOSLG  RET-HIIT: 3.77 ± 0.42 to 3.79 ± 0.48; Mean (95% CI) -0.02 (-0.14 to 0.11); p = NS  AET-HIIT: 3.73 ± 0.55 to 3.84 ± 0.46; Mean (95% CI) 0.08 (-0.06 to 0.23); p = NS  CSF-1  RET-HIIT: 6.73 ± 0.36 to 6.77 ± 0.41; Mean (95% CI) -0.01 (-0.16 to 0.13); p = NS  AET-HIIT: 6.83 ± 0.51 to 6.92 ± 0.45; Mean (95% CI) 0.08 (-0.04 to 0.20); p = NS  IFN-γ  RET-HIIT: 0.88 ± 1.59 to 1.01 ± 1.60; Mean (95% CI) 0.09 (-0.65 to 0.82); p = NS  AET-HIIT: 1.20 ± 0.98 to 1.46 ± 0.95; Mean (95% CI) 0.28 (-0.14 to 0.69); p = NS  IL-10  RET-HIIT: 0.92 ± 0.27 to 0.81 ± 0.26; Mean (95% CI) -0.08 (-0.28 to 0.13); p = NS  AET-HIIT: 0.95 ± 0.23 to 0.70 ± 0.18; Mean (95% CI) -0.29 (-0.40 to -0.19); p < 0.05  FasL  RET-HIIT: 5.18 ± 0.49 to 5.38 ± 0.58; Mean (95% CI) 0.11 (-0.05 to 0.27); p = NS  AET-HIIT: 5.06 ± 0.64 to 5.38 ± 0.53; Mean (95% CI) 0.31 (0.13 to 0.48); p < 0.05  CXCL9  RET-HIIT: 6.21 ± 0.73 to 6.73 ± 0.84; Mean (95% CI) 0.48 (0.13 to 0.83); p < 0.05  AET-HIIT: 6.50 ± 1.03 to 7.13 ± 0.83; Mean (95% CI) 0.66 (0.32 to 1.00); p < 0.05  MIC A/B  RET-HIIT: 3.11 ± 1.07 to 3.06 ± 1.04; Mean (95% CI) -0.06 (-0.19 to 0.06); p = NS  AET-HIIT: 2.84 ± 1.16 to 2.88 ± 1.16; Mean (95% CI) 0.02 (-0.09 to 0.14); p = NS | *Inflammation Biomarkers*  CD40-L  6.52 ± 1.33 to 4.82 ± 1.58; Mean (95% CI) -1.73 (-2.47 to -0.99); p < 0.05  EGF  8.16 ± 1.46 to 6.39 ± 1.53; Mean (95% CI) -1.77 (-2.55 to -1.00); p < 0.05  IL-6  2.59 ± 1.01 to 3.11 ± 1.01; Mean (95% CI) 0.49 (0.12 to 0.87); p < 0.05  TRAIL  7.02 ± 0.51 to 7.21 ± 0.42; Mean (95% CI) 0.18 (0.03 to 0.33); p < 0.05  CD8a  7.99 ± 0.72 to 8.09 ± 0.80; Mean (95% CI) 0.10 (-0.10 to 0.30); p=NS  DCN  3.44 ± 0.54 to 3.62 ± 0.42; Mean (95% CI) 0.17 (0.03 to 0.30); p < 0.05  CCL17  7.56 ± 1.07 to 6.84 ± 1.11; Mean (95% CI) -0.73 (-1.22 to -0.24); p < 0.05  CASP-8  3.95 ± 1.01 to 3.03 ± 0.86; Mean (95% CI) -0.96 (-1.39 to -0.53); p < 0.05  ICOSLG  3.65 ± 0.49 to 3.84 ± 0.43; Mean (95% CI) 0.18 (0.05 to 0.31); p < 0.05  CSF-1  6.68 ± 0.49 to 6.86 ± 0.45; Mean (95% CI) 0.17 (0.08 to 0.27); p < 0.05  IFN-γ  0.88 ± 1.65 to 1.49 ± 1.25; Mean (95% CI) 0.57 (-0.05 to 1.19); p = NS  IL-10  0.92 ± 0.26 to 0.71 ± 0.22; Mean (95% CI) -0.27 (-0.41 to -0.12); p < 0.05  FasL  5.06 ± 0.60 to 5.42 ± 0.63; Mean (95% CI) 0.33 (0.16 to 0.51); p < 0.05  CXCL9  6.29 ± 0.95 to 6.82 ± 0.94; Mean (95% CI) 0.47 (0.10 to 0.84); p < 0.05  MIC A/B  2.97 ± 1.17 to 3.06 ± 1.29; Mean (95% CI) 0.09 (-0.03 to 0.21); p = NS | *Inflammation Biomarkers*  Mean difference, compared to CG  CD40-L  RET-HIIT: 0.19 (−0.60 to 0.99); p = NS  AET-HIIT: 0.74 (−0.05 to 1.53); p = NS  EGF  RET-HIIT: 0.29 (−0.49 to 1.07); p = NS  AET-HIIT: 0.75 (−0.03 to 1.52); p = NS  IL-6  RET-HIIT: −0.47 (−0.87 to −0.07); p<0.05  AET-HIIT: −0.15 (−0.55 to 0.25); p = NS  TRAIL  RET-HIIT: −0.17 (−0.38 to 0.03); p = NS  AET-HIIT: 0.05 (−0.16 to 0.26); p = NS  CD8a  RET-HIIT: −0.28 (−0.57 to 0.004); p = NS  AET-HIIT: −0.20 (−0.48 to 0.09); p = NS  DCN  RET-HIIT: −0.14 (−0.31 to 0.03); p = NS  AET-HIIT: 0.005 (−0.16 to 0.17); p = NS  CCL17  RET-HIIT: 0.15 (−0.44 to 0.74); p = NS  AET-HIIT: 0.40 (−0.19 to 0.99); p = NS  CASP-8  RET-HIIT: 0.20 (−0.25 to 0.66); p = NS  AET-HIIT: 0.40 (−0.05 to 0.86); p = NS  ICOSLG  RET-HIIT: −0.14 (−0.32 to 0.04); p = NS  AET-HIIT: −0.05 (−0.23 to 0.13); p = NS  CSF-1  RET-HIIT: −0.13 (−0.30 to 0.03); p = NS  AET-HIIT: −0.03 (−0.19 to 0.13); p = NS  IFN-γ  RET-HIIT: −0.51 (−1.20 to 0.17); p = NS  AET-HIIT: −0.14 (−0.82 to 0.54); p = NS  IL-10  RET-HIIT: 0.13 (−0.02 to 0.29); p = NS  AET-HIIT: −0.0004 (−0.14 to 0.14); p = NS  FasL  RET-HIIT: −0.15 (−0.39 to 0.08); p = NS  AET-HIIT: −0.02 (−0.25 to 0.21); p = NS  CXCL9  RET-HIIT: 0.04 (−0.37 to 0.45); p = NS  AET-HIIT: 0.28 (−0.13 to 0.69); p = NS  MIC A/B  RET-HIIT: −0.14 (−0.30 to 0.03); p = NS  AET-HIIT: −0.06 (−0.22 to 0.11); p = NS |
| Irwin et al.  *Cancer Epidemiol Biomarkers Prev.*  2009 | N = 68  IG: 36  CG: 32  *Mean Age (years)*  IG: 56.4±9.5  CG: 55.6±7.7  *BMI (kg/m2)*  IG: 30.4±6.0  CG: 30.1±7.4 | Stage 0-IIIA breast cancer  *In Situ*  IG: 11%  CG: 13%  *I*  IG: 56%  CG: 25%  *II*  IG: 25%  CG: 44%  *IIIA*  IG: 8%  CG: 19% | *Chemotherapy*  IG: 19%  CG: 22%  *Radiotherapy*  IG: 42%  CG: 22%  *Chemotherapy*  and *Radiotherapy*  IG: 33%  CG: 41%  *Hormonal Therapy*  IG: 59%  CG: 72% | *Type of activity:* AET  *Exercise modality:* walking activity, although participants could choose to meet the exercise goal through other  forms of aerobic activity  *Protocol duration:* 5 weeks  *Frequency:* 3 (week 1) to 5 (week 5) session/wk  *Volume (session):* 15 min (week 1) to 30 min (week 5)  *Intensity:* moderate-intensity  *Supervised and home-based*  *After chemotherapy and/or radiotherapy treatments* | Usual care | NR | NR | *Metabolism Biomarkers*  Fasting insulin (μU/mL)  IG mean change: -1.75 ± 2.32 vs CG mean change: 3.49 ± 2.46; p = 0.089  IGF-1 (ng/mL)  IG mean change: -7.36 ± 6.02 vs CG mean change: +12.70 ± 6.39; p = 0.026  IGFBP-3 (μg/mL)  IG mean change: -0.19 ± 0.08 vs CG mean change: +0.15 ± 0.10; p = 0.006 |
| Jones et al.  *Cancer Prev Res (Phila).*  2013 | N = 67  IG: 36  CG: 31  *Mean Age (years)*  IG: 56.4±9.6  CG: 55.4±7.6  *BMI (kg/m2)*  IG: 30.6±6.0  CG: 29.4±7.3 | Stage 0-IIIA breast cancer  *In Situ*  IG: 4 (11%)  CG: 4 (13%)  *I*  IG: 20 (56%)  CG: 7 (23%)  *II*  IG: 9 (25%)  CG: 14 (45%)  *III*  IG: 3 (8%)  CG: 6 (19%) | *Chemotherapy*  IG: 7 (19%)  CG: 7 (23%)  *Radiotherapy*  IG: 15 (42%)  CG: 7 (23%)  *Chemotherapy*  and *Radiotherapy*  IG: 12 (33%)  CG: 13 (42%)  *Hormonal Therapy*  IG: 21 (59%)  CG: 22 (71%) | *Type of activity:* AET  *Exercise modality:* brisk walking, though participants could meet the exercise goal through other forms of aerobic exercise (stationary biking and elliptical training)  *Protocol duration:* 5 weeks  *Frequency:* 5 session/wk  *Volume (session):* 15 min (week 1) to 30 min (week 5)  *Intensity:* 50% predicted HR to 60-80% predicted HR  *Supervised and home-based*  *After chemotherapy and/or radiotherapy treatments* | Usual care | NR | NR | *Inflammation Biomarkers*  IL-6 (pg/mL)  IG: 3.59 ± 6.03 vs CG: 1.91 ± 1.19; p = 0.91  CRP (mg/L)  IG: 2.39 ± 2.26 vs CG: 2.23 ± 2.60; p = 0.73  TNF-α (pg/mL)  IG: 1.17 ± 0.40 vs CG: 1.35 ± 0.63; p = 0.54 |
| Janelsins et al.  *Clin Breast Cancer.*  2011 | N = 19  IG: 9  CG: 10  *Mean Age (years)*  IG: 54.33±10.64  CG:  52.70±6.67  *BMI (kg/m2)*  IG: 24.89±5.78  CG: 24.97±4.39 | Stage 0-IIIB breast cancer | *Lumpectomy*  IG: 55.60%  CG: 60.00%  *Unilateral Mastectomy*  IG: 33.30%  CG: 30.00%  *Bilateral Mastectomy*  IG: 11.10%  CG: 10.00%  *Chemotherapy*  IG: 66.70%  CG: 33.30%  *Radiotherapy*  IG: 100%  CG: 90.00% | *Type of activity:* tai chi chuan exercise  *Exercise modality:* warm-up, Yang-style tai chi chuan using the 15-move short form, and guided breathing, imagery, and meditation  *Protocol duration:* 12 weeks  *Frequency:* 3 session/wk  *Volume (session):* 60 min: 10 min warm-up, 40 min of tai chi chuan exercise, 10 min of guided breathing, imagery, and meditation  *Intensity:* /  *Supervised*  *After chemotherapy and/or radiotherapy treatments* | Psychosocial support  therapy | *Inflammation Biomarkers*  IL-6 (pg/mL): mean values = 2.63 ± 3.96 vs. 4.63 ± 6.97; mean change = 2.00 ± 5.53; p = NR  IL-2 (pg/mL): mean values = 12.48 ± 22.15 vs. 3.66 ± 5.83; mean change = -8.82 ± 23.41; p = NR  IFN-γ (pg/mL): mean values = 1.34 ± 2.42 vs. 1.17 ± 1.78; mean change = 0.17 ± 3.56; p = NR  *Metabolism Biomarkers*  IGF-1 (ng/mL): mean values = 156.81 ± 19.58 vs. 129.49 ± 43.83; mean change = -27.32 ± 45.07; p = NR  IGFBP-1 (ng/mL): mean values = 72.64 ± 25.64 vs. 76.40 ± 42.76; mean change = 3.76 ± 27.32; p = NR  IGFBP-3 (ng/mL): mean values = 39.22 ± 6.26 vs. 40.11 ± 7.29; mean change = 0.89 ± 3.12; p = NR  Fasting insulin (μIU/mL): mean values = 15.34 ± 5.36 vs. 16.75 ± 7.99; mean change = 1.41 ± 7.05; p = NR | *Inflammation Biomarkers*  IL-6 (pg/mL): mean values = 2.44 ± 1.79 vs. 2.42 ± 1.74; mean change = -0.02 ± 1.51; p = NR  IL-2 (pg/mL): mean values = 3.73 ± 4.96 vs. 8.32 ± 13.22; mean change = 4.59 ± 12.64; p = NR  IFN-γ (pg/mL): mean values = 7.79 ± 9.70 vs. 10.21 ± 15.55; mean change = 2.42 ± 6.45; p = NR  *Metabolism Biomarkers*  IGF-1 (ng/mL): mean values = 111.76 ± 82.64 vs. 95.12 ± 58.65; mean change = -16.64 ± 66.5; p = NR  IGFBP-1 (ng/mL): mean values = 92.22 ± 39.02 vs. 101.34 ± 50.01; mean change = 9.12 ± 36.44; p = NR  IGFBP-3 (ng/mL): mean values = 40.81 ± 13.55 vs. 40.11 ± 15.13; mean change = -0.70 ± 3.77; p = NR  Fasting insulin (μIU/mL): mean values = 15.83 ± 9.10 vs. 30.85 ± 29.86; mean change = 15.02 ± 23.62; p = NR | *Metabolism and Inflammation Biomarkers*  IL-6 (pg/mL): mean change = IG: 2.00 ± 5.53 vs. CG: 0.02 ± 1.51; p = 0.297  IL-2 (pg/mL): mean change = IG: 8.82 ± 23.41 vs. CG: 4.59 ± 12.64; p = 0.369  IFN-γ (pg/mL): mean change = IG: 0.17 ± 3.56 vs. CG: 2.42 ± 6.45; p = 0.831  *Metabolism Biomarkers*  IGF-1 (ng/mL): mean change = IG: -27.32 ± 45.07 vs. CG: -16.64 ± 66.5; p = 0.495  IGFBP-1 (ng/mL): mean change = IG: 3.76 ± 27.32 vs. CG: 9.12 ± 36.44; p = 0.749  IGFBP-3 (ng/mL): mean change = IG: 0.89 ± 3.12 vs. CG: -0.70 ± 3.77; p = 0.299  Fasting insulin (μIU/mL): mean change = IG: 1.41 ± 7.05 vs. CG: 15.02 ± 23.62; p = 0.099 |
| Kim et al.  *PLoS One.*  2017 | N = 24  IG: 11  CG: 13  *Mean Age (years)*  N = 52.4±6.5  IG: 56.0±6.5  CG: 49.3±4.8  *BMI (kg/m2)*  IG: 23.9±2.7  CG: 25.0±4.7 | Stage I-III breast cancer | *Lumpectomy*  IC: 9 (81.8%)  CG: 11 (84.6%)  *Mastectomy*  IC: 2 (18.2%)  CG: 2 (15.4%)  *Chemotherapy*  IC: 9 (81.8%)  CG: 12 (92.3%)  *Radiotherapy*  IC: 10 (90.9%)  CG: 12 (92.3%) | *Type of activity:* CET  *Exercise modality:* warm-up (stretching and flexibility), step aerobics (AET) using a platform followed by strength training (RET) (shoulder press, black burn exercise, wall push-up, biceps curl-up, plank exercise, leg bridge, squat, calf raise) and cool down  *Protocol duration:* 12 weeks  *Frequency:* 3 session/wk  *Volume (session):* 10 min of warm up; 20 min of AET + 1-3 sets of 12-16 reps of RET; 10 min of cool-down  *Intensity:* 11-13 RPE (at the start) to 13-15 RPE (at the end)  *Supervised*  *After chemotherapy and/or radiotherapy treatments* | No intervention | *Metabolism Biomarkers*  Fasting insulin (uIU)  11.2 (6.1) vs 7.6 (3.0); p = 0.018  *Inflammation Biomarkers*  CRP (mg/mL)  1.18 (1.37) vs 0.71 (0.68); p= 0.305  Leptin (ng/mL)  18.7 (16.1) vs 14.9 (15.5); p= 0.022  Adiponectin (μg/mL)  7.46 (2.77) vs 7.92 (3.14); p= 0.248  *Tumor-metabolism Biomarkers*  DKK1 (pg/ml)  2571 ± 151 vs 1716 ± 145; p = 0.002  SFRP1  880 ± 119 vs 468 ± 75; p = 0.008 | *Metabolism Biomarkers*  Fasting Fasting insulin (uIU)  10.4 (7.5) vs 11.2 (9.5); p= 0.734  *Inflammation Biomarkers*  CRP (mg/mL)  1.60 (2.89) vs 1.43 (1.53); p= 0.431  Leptin (ng/mL)  10.9 (7.2) vs 12.5 (12.6); p= 0.377  Adiponectin (μg/mL)  6.45 (3.75) vs 6.37 (3.87); p= 0.507  *Tumor-metabolism Biomarkers*  DKK1 (pg/ml)  2613 ± 203 vs 2365 ± 197; p = 0.08  SFRP1  1,085 ± 149 vs 1,039 ± 110; p = 0.35 | NR |
| Kirkham et al.  *Breast Cancer Res Treat.*  2017 | N = 24  IG: 13  CG: 11  *Mean Age (years)*  N = 50±9  IG: 51±9  CG: 50±10  *BMI (kg/m2)*  IG: 25.0±4.8  CG: 26.7±5.1 | Stage I–III breast cancer  *I*  IG: 1 (8%)  CG: 3 (27%)  *II*  IG: 7 (54%)  CG: 5 (45%)  *III*  IG: 5 (38%)  CG: 3 (27%) | *Chemotherapy*  100% (doxorubicin) | *Type of activity:* AET  *Exercise modality:* warm-up, AET, and cool-down.  *Protocol duration:* /  *Frequency:* 1 session 24 h prior to each doxorubicin treatment  *Volume (session):* 10 min of warm-up; 30 min of AET; 5 min of cool-down  *Intensity:* 70% HRR  *Supervised*  *During cancer treatments* | No intervention | *Cardiac Biomarkers*  cTnT (pg/mL)  1.3 ± 2.1 vs 13.6 ± 11.2; p < 0.05  NT-proBNP (pg/mL)  52 ± 30 vs 106 ± 78; p < 0.05 | *Cardiac Biomarkers*  cTnT (pg/mL)  1.5 ± 2.3 vs 11.6 ± 8.3; p < 0.05  NT-proBNP (pg/mL)  59 ± 35 vs 77 ± 39; p < 0.05 | *Cardiac Biomarkers*  Hemoglobin (g/dl)  IG Pre: 13.0 ± 1.0, Change: − 2.3 ± 1.0 vs CG Pre: 12.7 ± 1.1, Change: − 2.5 ± 1.5; p > 0.05 |
| Lahart et al.  *BMC Cancer.*  2016 | N = 80  IG: 40  CG: 40  *Mean Age (years)*  N = 53.6±9.4  IG: 52.4±10.3  CG: 54.7±8.3  *BMI (kg/m2)*  *BMI ≥ 30*  N = 22 (28%)  *BMI 25–29.9*  N = 29 (36%)  *BMI <25*  N = 29 (36%) | Stage I–III breast cancer | NR | *Type of activity:* AET  *Exercise modality:* NR  *Protocol duration:* 6 months  *Frequency:* 3-5 (months 1-3) to 5-7 (months 4-6) session/wk  *Volume (session):* 30 min of AET  *Intensity:* moderate-intensity  *Home-based*  *After chemotherapy and/or radiotherapy treatments* | No intervention | NR | NR | *Metabolism Biomarkers*  Total Cholesterol (mmol∙L−1)  Mean change: -0.45 (-0.71 to -0.18); p=0.001  HDL (mmol∙L−1)  Mean change: -0.06 (-0.15 to 0.04); p=0.264  LDL (mmol∙L−1)  Mean change: -0.30 (-0.56 to -0.04); p=0.023  Triglycerides (mmol∙L−1)  Mean change: -0.10 (-0.27 to -0.07); p=0.240  Glucose (mmol∙L−1)  Mean change: -0.07 (-0.38 to 0.25); p=0.683  Fasting insulin (pmol∙L−1)  Mean change: 8.31 (-12.31 to 28.95); p=0.425  HOMA  Mean change: 0.42 (-0.88 to 1.73); p=0.520 |
| Lee et al.  *JAMA Oncol.*  2019 | N = 100  IG: 50  CG: 50  *Mean Age (years)*  N = 53.5±10.4  IG: 53 (38, 68 IQR)  CG: 52 (37, 66 IQR)  *BMI (kg/m2)*  N =  33.5±5.5  IG: 33 (30, 36 IQR)  CG: 32 (30, 35 IQR) | Stage I-III breast cancer  *I*  IG: 20 (40%)  CG: 21 (42%)  *II*  IG: 19 (38%)  CG: 19 (38%)  *III*  IG: 11 (22%)  CG: 10 (20%) | *Chemotherapy*  IG: 8 (16%)  CG: 5 (10%)  *Radiotherapy*  IG: 4 (8%)  CG: 6 (12%)  *Chemotherapy*  and *Radiotherapy*  IG: 38 (76%)  CG: 39 (78%)  *Hormonal Therapy*  IG: 44 (88%)  CG: 34 (69%) | *Type of activity:* CET  *Exercise modality:* 2 sessions of CET and 1 session of only AET. AET = treadmill walking, machine rowing, or cycling; RET = leg presses, leg flexions or extensions, chest presses, seated rows, biceps curls, and triceps pulldowns  *Protocol duration:* 16 weeks  *Frequency:* 3 session/wk  *Volume (session):* 50 min of AET; 80 min of CET  *Intensity:* AET = 65%-80% HRmax; RET = 60-80% 1RM  *Supervised*  *After chemotherapy and/or radiotherapy treatments* | No intervention | *Metabolism Biomarkers*  HDL (mg/dL)  43.1 ± 6.6 vs 64.7 ± 7.8; p = 0.001  LDL (mg/dL)  167.9 ± 19.7 vs 119.3 ± 12.1; p<0.001 | *Metabolism Biomarkers*  HDL (mg/dL)  41.0 ± 4.3 vs 39.9 ± 4.0; p = 0.45  LDL-C (mg/dL)  172.4 ± 20.3 vs 178.3 ± 21.7; p = 0.59 | *Metabolism Biomarkers*  HDL (mg/dL)  Mean difference: 24.4 (27.9 to 17.9); p = 0.001  LDL (mg/dL)  Mean difference: −48.6 (−61.2 to −27.6); p = 0.001 |
| Lee et al.  S*ci Rep.* 2020 | N = 30  IG: 15  CG: 15  *Mean Age (years)*  N = 46.9±9.8  IG: 49.1±7.9  CG: 44.7±11.2  *BMI (kg/m2)*  N = 31.6±7.7  IG: 33.1±7.6  CG: 30.1±7.7 | Stage I-III breast cancer  *I*  IG: 1 (6%)  CG: 1 (6%)  *II*  IG: 5 (30%)  CG: 4 (24%)  *III*  IG: 9 (64%)  CG: 10 (70%) | *Chemotherapy*  IG: 15 (100%)  CG: 15 (100%) | *Type of activity:* HIIT  *Exercise modality:* stationary bicycle  *Protocol duration:* 8 weeks  *Frequency:* 3 session/wk  *Volume (session):* 5minute warm-up, 20-minute HIIT protocol  *Intensity:* 1 minute high-intensity exercise (90% of PPO) followed by 2 minutes of low intensity (10% of PPO)  *Supervised*  *During cancer treatments* | No intervention | *Cardiac Biomarkers*  MMP-1 (ng/ml)  14.9 ± 11.7 ± 19.4 ± 12.4; p = 0.17  MMP-2 (ng/ml)  76.6 ± 11.2 ± 83.2 ± 13.1; p = 0.007  MMP-7 (ng/ml)  4.4 ± 0.9 ± 4.3 ± 0.8; p = 0.85  MMP-9 (ng/ml)  104.3 ± 51.9 ± 65.2 ± 69.1; p = 0.01  MMP-10 (ng/ml)  1.4 ± 0.7 ± 1.4 ± 0.5; p = 0.91  TIMP-1 (ng/ml)  362.2 ± 92.5 ± 377.5 ± 91.5; p = 0.65  TIMP-2 (ng/ml)  93.3 ± 12.6 ± 93.7 ± 12.1; p = 0.92 | *Cardiac Biomarkers*  MMP-1 (ng/ml)  14.3 ± 19.2 ± 18.8 ± 21.6; p = 0.14  MMP-2 (ng/ml)  69.0 ± 8.9 ± 77.6 ± 11.1; p = 0.003  MMP-7 (ng/ml)  4.8 ± 2.6 ± 4.3 ± 2.7; p = 0.13  MMP-9 (ng/ml)  115.5 ± 47.2 ± 90.4 ± 67.9; p = 0.10  MMP-10 (ng/ml)  1.7 ± 1.4 ± 1.5 ± 0.9; p = 0.62  TIMP-1 (ng/ml)  322.3 ± 92.6 ± 354.3 ± 87.3; p = 0.17  TIMP-2 (ng/ml)  86.3 ± 10.9 ± 89.8 ± 14.5; p = 0.11 | *Cardiac Biomarkers*  MMP-1 (ng/ml)  Mean difference: 4.5 (−2.1 to 11.1); p = 0.98  MMP-2 (ng/ml)  Mean difference: 6.5 (2.1 to 10.9); p = 0.53  MMP-7 (ng/ml)  Mean difference: −0.05 (−0.64 to 0.54); p = 0.33  MMP-9 (ng/ml)  Mean difference: −46.4 (−80.4 to 12.4); p = 0.65  MMP-10 (ng/ml)  Mean difference: 0.02 (−0.4 to 0.5); p = 0.61  TIMP-1 (ng/ml)  Mean difference: 15.3 (−54.4 to 84.9); p = 0.67  TIMP-2 (ng/ml)  Mean difference: 0.4 (−7.2 to 7.9); p = 0.46 |
| Ligibel et al.  *J Clin Oncol.*  2008 | N = 100  IG: 51  CG: 49  *Mean Age (years)*  IG: 52±9  CG: 53±9  *BMI (kg/m2)*  IG: 30.3±5.9  CG: 31.4±6.8 | Stage I-III breast cancer  *I*  IG: 22 (43%)  CG: 21 (43%)  *II*  IG: 22 (43%)  CG: 22 (44%)  *III*  IG: 6 (12%)  CG: 4 (8%)  *NR*  IG: 0 (0%)  CG: 2 (4%) | *Lumpectomy*  IG: 32 (63%)  CG: 27 (55%)  *Mastectomy*  IG: 19 (37%)  CG: 20 (41%)  *Axillary surgery only*  IG: 0 (0%)  CG: 1 (2%)  *Surgery NR:*  IG: 0 (0%)  CG: 1 (2%)  *Chemotherapy*  IG: 39 (76%)  CG: 33 (67%)  *Hormonal Therapy*  IG: 37 (73%)  CG: 30 (61%) | *Type of activity:* CET  *Exercise modality:* supervised RET + home-based AET.  RET = Warm-up (walking on treadmill and stretching), strength training (leg press, quadriceps extension, hamstring curl, hip adductor, hip abductor, abdominal crunches, calf press, and leg lifts); AET = cardiovascular exercise (walking)  *Protocol duration:* 16 weeks  *Frequency:* 2 sessions/wk of RET (supervised) + self-selected number of sessions of AET (home-based)  *Volume (session):* RET = 15 min warm-up, 2-4 sets of 10 reps for each muscle group; AET = 90 min of AET on their own each week  *Intensity:* RET = 80% 1RM; AET = 55-80% HRmax  *Supervised and home-based*  *After chemotherapy and/or radiotherapy treatments* | No intervention | *Metabolism Biomarkers*  Fasting insulin (μg/mL): mean values = 10.3±10.4 vs. 7.5±6.6; mean change = -2.9±8.0; p = 0.03  Glucose (mg/dL): mean values = 85.8±18.6 vs. 82.9±16.8; mean change = -3.1±19.4; p = NS  HOMA-IR: mean values = 2.4±2.9 vs. 1.7±1.6; mean change = -0.8±2.5; p = 0.05 | *Metabolism Biomarkers*  Fasting insulin (μg/mL): mean values = 8.7±6.4 vs. 8.4±6.1; mean change = -0.3±3.8; p = NS  Glucose (mg/dL): mean values = 88.2±13.5 vs. 87.8±15.7; mean change = -0.4±12.8; p = NS  HOMA-IR: mean values = 2.0±1.5 vs. 1.9±1.6; mean change = -0.04±1.1; p = NS | *Metabolism Biomarkers*  Fasting insulin (μg/mL): mean values = IG: 7.5±6.6 vs. CG: 8.4±6.1; p = 0.07  Glucose (mg/dL): mean values = IG: 82.9±16.8 vs. CG: 87.8±15.7; p = 0.47  HOMA-IR: mean values = IG: 1.7±1.6 vs. CG: 1.9±1.6; p = 0.09 |
| Mijwel et al.  *FASEB J*  2018 | N = 23  AT-HIIT: 6  RT-HIIT: 7  CG: 10  *Mean Age (years)*  AT-HIIT: 51.5±7.0  RT-HIIT: 54.3±11.0  CG: 51.0±13.1  *BMI (kg/m2)*  NR | Stage I-IIIa breast cancer | *Chemotherapy*  AT-HIIT: 6 (100%)  RT-HIIT: 7 (100.%)  CG: 10 (100%) | RT-HIIT:  *Type of activity:* RET-HIIT  *Exercise modality:* RET targeting the major muscle groups, followed by HIIT on a cycle ergometer  *Protocol duration:* 16 weeks  *Frequency:* 2 session/wk  *Volume (session):* 8–12 Rep + 3×3 min bouts of aerobic HIIT  *Intensity:* 70-80% of 1RM (RET) and 16–18 RPE (Borg 6–20) for HIIT  *Supervised*  *During cancer treatments*  AT–HIIT:  *Type of activity:* AET-HIIT  *Exercise modality:* moderate intensity continuous AET on cycle ergometer, elliptical ergometer, or a treadmill, followed by aerobic HIIT on a cycle ergometer  *Protocol duration:* 16 weeks  *Frequency:* 2 session/wk  *Volume (session):* 20 minutes (AET) + 3×3 min bouts of aerobic HIIT  *Intensity:* 13–15 RPE (Borg 6–20) for AT and 16–18 RPE (Borg 6–20) for HIIT  *Supervised*  *During cancer treatments* | Usual care | *Metabolic Biomarkers*  CS activity:  AET–HIIT = NR; p > 0.05  RET-HIIT = NR; p > 0.05  Oxphos complexes:  AET–HIIT = Complex IV: NR; p = 0.04  RET-HIIT = NR; p > 0.05  MHC isoforms:  AET–HIIT = NR; p > 0.05  RET-HIIT = NR; p > 0.05  Mitophagy:  PINK1:  AET–HIIT = NR; p > 0.05  RET-HIIT = NR; p > 0.05 | *Metabolic Biomarkers*  CS activity: NR; p < 0.05  Oxphos complexes: NR; p > 0.05  MHC isoforms: type I: NR; p = 0.006  Mitophagy:  PINK1: NR; p = 0.031  Antioxindant capacity:  SOD2: NR; p = 0.005 | *Metabolic Biomarkers*  CS activity:  CG vs. AET-HIIT = p = 0.005  CG vs. RET-HIIT = p = 0.027  Oxphos complexes:  AET–HIIT vs CG = complex I: p = 0.003; complex II:  p = 0.007; complex IV: p = 0.004  AET-HIIT vs. RET-HIIT = complex I:  p = 0.011; complex II: p = 0.005; complex IV: p = 0.002  MHC isoforms:  RET-HIIT vs. CG = MHC type I: p = 0.016  Mitophagy:  PINK1:  AET–HIIT vs. CG = NR; p = 0.012 |
| Pagola et al.  *Int J Sports Med.*  2020 | N = 23  IG: 13  CG: 10  *Mean Age (years)*  N = 50±8  IG: 47±7  CG: 51±6  *BMI (kg/m2)*  IG: 25.8±5.8  CG: 27.3±3.7 | Breast cancer | *Chemotherapy*  IG: 50%  CG: 87%  *Radiotherapy*  IG: 23%  CG: 40%  *Hormonal Therapy*  IG: 92%  CG: 100% | *Type of activity:* high-intensity training  *Exercise modality:* dynamic warm-up (respiratory and  mobility exercises) followed by high-intensity aerobic (cycle-ergometer pedaling, aerobic games, treadmill running, or elliptical ergometer) and resistance exercises (elastic bands, dumbbells, Fit Balls, suspension training, bodyweight exercises [biceps, triceps, chest, deltoid, back and abdominal muscles, quadriceps, hamstrings, buttocks, calf, and adductor/abductor])  *Protocol duration:* 16 weeks  *Frequency:* 2 session/wk  *Volume (session):* 10 min warm-up; AET = 35 min divided into 3 consecutive periods of 10–15 min; RET = 8–10 exercises/session, 2-3 sets of 8-12 reps  *Intensity:* AET = 7–8 RPE (1-10); RET = 6-7 RPE (1-10)  *Supervised*  *After chemotherapy and/or radiotherapy treatments* | *Type of activity:* moderate-intensity training  *Exercise modality:* the same modality of high-intensity training group  *Protocol duration:* 16 weeks  *Frequency:* 2 session/wk  *Volume (session):* the same volume of high-intensity training group  *Intensity:* AET = 6 RPE (1-10); RET = 6-7 RPE (1-10);  *Supervised RET + not supervised AET*  *After cancer treatments* | *Inflammation Biomarkers*  NLR ratio: mean values = 2.0±0.8 vs. 1.6±0.6; mean change = −0.4 (−0.7, −0.1); p = 0.028 | *Inflammation Biomarkers*  NLR ratio: mean values = 2.3±0.6 vs. 2.2±1.3; mean change = −0.1 (−0.9, 0.7); p = 0.646 | *Inflammation Biomarkers*  NLR ratio: mean values = IG: 1.6±0.6 vs. CG: 2.2±1.3; mean change = −0.3 (−1.1, 0.7); p = 0.595 |
| Schmitz et al.  *Cancer Epidemiol Biomarkers Prev.*  2005 | N = 81  IG: 40  CG: 41  *Mean Age (years)*  IG: 53.3±8.7  CG: 52.8±7.6  *BMI (kg/m2)*  IG: 25.94±0.73  CG: 25.76±0.73 | Stage I-III breast cancer  *DCIS*  IG: 7 (18%)  CG: 5 (12%)  *I*  IG: 16 (43%)  CG: 16 (39%)  *II*  IG: 13 (34%)  CG: 18 (44%)  *III*  IG: 2 (5%)  CG: 2 (5%) | *Axillary dissection*  IG: 33 (87%)  CG: 39 (95%)  *Chemotherapy*  IG: 25 (66%)  CG: 30 (73%)  *Radiotherapy*  IG: 25 (66%)  CG: 26 (65%)  *Hormonal Therapy*  IG: 33 (85%)  CG: 33 (80%) | *Type of activity:* RET  *Exercise modality:* Nine weight-training exercises were performed using variable resistance machines and free weights (chest, back, shoulders, arms, buttocks, hips, and thighs)  *Protocol duration:* 6 months  *Frequency:* 2 session/wk  *Volume (session):* 2-3 sets of 8-12 reps  *Intensity:* the heaviest weight they could lift  *Supervised and not supervised*  *After chemotherapy and/or radiotherapy treatments* | No intervention | *Metabolism Biomarkers*  Fasting insulin (microunits/mL): mean values = 7.66±0.69 vs. 8.21±0.69; mean change = 0.55±0.51; p = 0.79  Glucose (mg/d): mean values = 92.19±1.85 vs. 88.49±1.88; mean change = -3.70±1.71; p = 0.90  HOMA-IR (units): mean values = 1.74±0.19 vs. 1.79±0.19; mean change = 0.05±0.15; p = 1.00  IGF-I (ng/mL): mean values = 172.93±11.58 vs. 181.22±11.55; mean change = 8.29±6.26; p = 0.16  IGF-II (ng/mL): mean values = 898.01±34.92 vs. 871.77±34.85; mean change = -26.23±16.73; p = 0.02  IGFBP-1 (ng/mL): mean values = 36.86±2.88 vs. 34.73±2.86; mean change = -2.14±2.32; p = 0.36  IGFBP-2 (ng/mL): mean values = 421.72±29.51 vs. 449.63±29.42; mean change = 27.91±16.83; p = 0.30  IGFBP-3 (ng/mL): mean values = 4,339.66±133.17 vs. 4,356.20±132.66; mean change = 16.54±85.89; p = 0.32 | *Metabolism Biomarkers*  Fasting insulin (microunits/mL): mean values = 7.59±0.67 vs. 7.94±0.67; mean change = 0.35±0.49; p = NR  Glucose (mg/d): mean values = 91.73±1.82 vs. 88.35±1.85; mean change = -3.38±1.66; p = NR  HOMA-IR (units): mean values = 1.76±0.18 vs. 1.80±0.19; mean change = 0.05±0.14; p = NR  IGF-I (ng/mL): mean values = 194.26±11.40 vs. 190.30±11.41; mean change = -3.97±6.10; p = NR  IGF-II (ng/mL): mean values = 891.26±34.40 vs. 919.54±34.42; mean change = 28.28±16.31; p = NR  IGFBP-1 (ng/mL): mean values = 36.94±2.82 vs. 37.75±2.83; mean change = 0.81±2.26; p = NR  IGFBP-2 (ng/mL): mean values = 472.86±29.04 vs. 476.45±29.06; mean change = 3.58±16.42; p = NR  IGFBP-3 (ng/mL): mean values = 4,519.72±130.91 vs. 4,655.05±131.02; mean change = 135.33±83.79; p = NR | *Metabolism Biomarkers*  Fasting insulin (microunits/mL): mean values = IG: 8.21±0.69 vs. CG: 7.94±0.67; p = 0.46  Glucose (mg/d): mean values = IG: 88.49±1.88 vs. CG: 88.35±1.85; p = 0.17  HOMA-IR (units): mean values = IG: 1.79±0.19 vs. CG: 1.80±0.19; p = 0.78  IGF-I (ng/mL): mean values = IG: 181.22±11.55 vs. CG: 190.30±11.41; p = 0.64  IGF-II (ng/mL): mean values = IG: 871.77±34.85 vs. CG: 919.54±34.42; p = 0.02  IGFBP-1 (ng/mL): mean values = IG: 34.73±2.86 vs. CG: 37.75±2.83; p = 0.47  IGFBP-2 (ng/mL): mean values = IG: 449.63±29.42 vs. CG: 476.45±29.06; p = 0.89  IGFBP-3 (ng/mL): mean values = IG: 4,356.20±132.66 vs. 4,655.05±131.02; p = 0.03 |
| Sprod et al.  *J Cancer Surviv.*  2012 | N = 19  IG: 9  CG: 10  *Mean Age (years)*  IG: 54.33±3.55  CG: 52.70±2.11  *BMI (kg/m2)*  IG: 24.89±1.93  CG: 24.97±1.39 | Stage 0-IIIb breast cancer | *Lumpectomy*  IG: 5 (56%)  CG: 6 (60%)  *Mastectomy*  IG: 4 (44%)  CG: 4 (40%)  *Chemotherapy*  84%  *Radiotherapy*  61%  *Hormonal Therapy*  56% | *Type of activity:* tai chi chuan exercise  *Exercise modality:* warm-up, Yang-style tai chi chuan using the 15-move short form, and guided breathing, imagery, and meditation  *Protocol duration:* 12 weeks  *Frequency:* 3 session/wk  *Volume (session):* 60 min: 10 min warm-up, 40 min of tai chi, 10 min of guided breathing, imagery, and meditation  *Intensity:* /  *Supervised*  *After chemotherapy and/or radiotherapy treatments* | Standard support  therapy control | *Inflammation Biomarkers*  IL-6 (pg/mL): mean values = 2.63±1.32 vs. 4.63±2.32; mean change = 2.00±1.84; p = 0.31  IL-8 (pg/mL): mean values = 9.37±1.80 vs. 9.69±2.05; mean change = 0.32±1.25; p = 0.80  Cortisol (μg/dL): mean values = 21.52±2.91 vs. 23.50±3.77; mean change = 1.98±2.30; p = 0.42  *Metabolism Biomarkers*  IGF-1 (ng/mL): mean values = 156.81±19.58 vs. 129.49±14.61; mean change = 27.32±15.02; p = 0.11  IGFBP-1 (ng/mL): mean values = 72.64±8.55 vs. 76.40±14.25; mean change = 3.76±9.10; p = 0.69  IGFBP-3 (ng/mL): mean values = 39.22±2.09 vs. 40.11±2.43; mean change = 0.89±1.04; p = 0.42  Glucose (mg/dL): mean values = 80.00±4.83 vs. 85.78±4.39; mean change = 5.78±2.86; p = 0.08  Fasting insulin (μIU/mL): mean values = 15.34±1.79 vs. 16.75±2.66; mean change = 1.34±2.35; p = 0.56 | *Inflammation Biomarkers*  IL-6 (pg/mL): mean values = 2.44±0.56 vs. 2.42±0.55; mean change = −0.01±0.48; p = 0.98  IL-8 (pg/mL): mean values = 11.06±2.57 vs. 7.24±1.93; mean change = −3.82±2.52; p = 0.17  Cortisol (μg/dL): mean values = 26.04±2.08 vs. 28.69±2.52; mean change = 2.65±2.76; p = 0.36  *Metabolism Biomarkers*  IGF-1 (ng/mL): mean values = 111.76±26.15 vs. 95.12±18.55; mean change = −16.64±21.03; p = 0.45  IGFBP-1 (ng/mL): mean values = 92.22±12.34 vs. 101.34±15.82; mean change = 9.12±11.53; p = 0.45  IGFBP-3 (ng/mL): mean values = 40.81±4.28 vs. 40.11±4.79; mean change = −0.70±1.19; p = 0.57  Glucose (mg/dL): mean values = 85.50±4.95 vs. 89.00±11.41; mean change = 3.50±7.10; p = 0.63  Fasting insulin (μIU/mL): mean values = 15.83±2.88 vs. 30.85±9.44; mean change = 15.03±7.47; p = 0.08 | *Inflammation Biomarkers*  IL-6 (pg/mL): mean values = IG: 4.63±2.32 vs. CG: 2.42±0.55; p = 0.28  IL-8 (pg/mL): mean values = IG: 9.69±2.05 vs. CG: 7.24±1.93; p = 0.17  Cortisol (μg/dL): mean values = IG: 23.50±3.77 vs. CG: 28.69±2.52; p = 0.86  *Metabolism Biomarkers*  IGF-1 (ng/mL): mean values = IG: 129.49±14.61 vs. CG: 95.12±18.55; p = 0.69  IGFBP-1 (ng/mL): mean values = IG: 76.40±14.25 vs. CG: 101.34±15.82; p = 0.72  IGFBP-3 (ng/mL): mean values = IG: 40.11±2.43 vs. CG: 40.11±4.79; p = 0.33  Glucose (mg/dL): mean values = IG: 85.78±4.39 vs. CG: 89.00±11.41; p = 0.78  Fasting insulin (μIU/mL): mean values = IG: 16.75±2.66 vs. CG: 30.85±9.44; p = 0.10 |
| *Abbreviations* 1RM: 1-repetition maximum; ACSM: American College of Sports Medicine; AET: aerobic training; AET–HIIT: moderate-intensity aerobic training combined with high-intensity interval training; AT–HIIT: aerobic training combined with high-intensity interval training; BDNF: brain-derived neurotrophic factor; CG: Control Group; CRP: C reactive protein; CSF-1: macrophage colony stimulating factor 1; CET: combined training; cTnT: cardiac Troponin T; CXCL9: C-X-C motif chemokine 9; DCN: decorin; DKK1: Dickkpof-1; FasL: Fas antigen ligand; GPx: Glutathione Peroxidase; Hb: hemoglobin; HbA1c: glycosylated hemoglobin; HDL: high density lipoprotein; HIIT: High Intensity Interval Training; HOMA1-IR: homeostatic model assessment index 1 - insulin resistance; HOMA2-IR: homeostatic model assessment index 2 - insulin resistance; HR: heart rate; ICOSLG: ICOS ligand; IG: Intervention Group; IGF: insulin grow factor; IGFBP: insulin grow factor bindign protein; IL: interleukin; LDL: low density lipoprotein; MDA: Malondialdehyde; MIC A/B: MHC class I polypeptide-related sequence A/B; min: minutes; MMP: matrix metalloproteinases; NLR: neutrophil-to-lymphocyte ratio; NO: Nitric Oxide; NR: not reported; NS: not significant; NT-proBNP: N-terminal prohormone of brain natriuretic peptide; OMNI-RES: OMNI Resistance Exercise Scale; PPO: peak power output; RPE: rating of perceived exertion; Rep: repetitions; RET: Resistance Training; RET-HIIT: resistance exercise training combined with high-intensity interval training; RT-HIIT: resistance training combined with high-intensity interval training; SFRP1: secreted frizzled-related protein-1; TIMP: tissue inhibitor of matrix metalloproteinases; TNF-a: tumor necrosis factor alpha; TRAIL: TNF-related apoptosis-inducing ligand; UK: United Kindom; USA: United States of America; VO2max: maximal oxygen consumption; wk: week. | | | | | | | | |
